# Interfacial Engineering of a Z-Scheme Bi_2_O_2_S/NiTiO_3_ Heterojunction Photoanode for the Degradation of Sulfamethoxazole in Water

**DOI:** 10.1021/acsami.4c20102

**Published:** 2024-12-05

**Authors:** Kehinde D. Jayeola, Dimpo S. Sipuka, Tsholofelo I. Sebokolodi, Jonathan O. Babalola, Minghua Zhou, Frank Marken, Omotayo A. Arotiba

**Affiliations:** † Department of Chemical Sciences, University of Johannesburg, Doornfontein Campus, Johannesburg 2028, South Africa; ‡ Centre for Nanomaterials Science Research, University of Johannesburg, Johannesburg 2028, South Africa; § Department of Chemistry, University of Ibadan, Ibadan 200005, Oyo State, Nigeria; ∥ Bowen University, Iwo 232101, Osun State, Nigeria; ⊥ Tianjin Key Laboratory of Environmental Technology for Complex Trans-Media Pollution, College of Environmental Science and Engineering, Nankai University, Tianjin 300350, China; # Department of Chemistry, University of Bath, Claverton Down, Bath BA2 7AY, U.K.

**Keywords:** photoelectrocatalytic oxidation, Z-scheme, NiTiO_3_, Bi_2_O_2_S, heterojunction photoanode, sulfamethoxazole

## Abstract

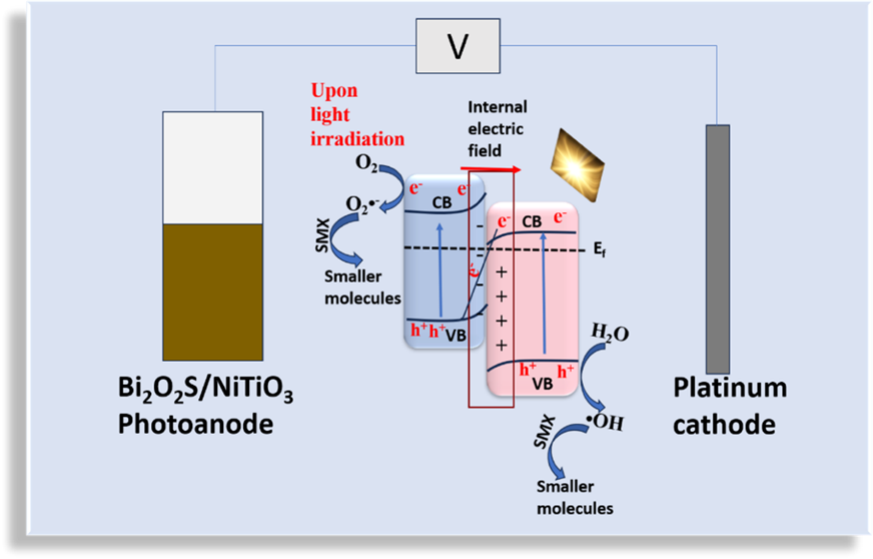

To develop a semiconductor interface with enhanced spatial separation of carriers under visible light irradiation for the photoelectrochemical (PEC) oxidation process, we explored the fabrication of a Bi_2_O_2_S/NiTiO_3_ heterojunction photoanode for the removal of sulfamethoxazole in water. The Bi_2_O_2_S/NiTiO_3_ photoanode was synthesized via an in situ hydrothermal process, and it exhibited better light absorption and charge separation, as well as a reduced rate of recombination of photoexcited charge species compared to pristine Bi_2_O_2_S and NiTiO_3._ The improved photoelectrocatalytic performance was attributed to the synergistic interaction between Bi_2_O_2_S and NiTiO_3_ and the presence of an S–O bond at the heterojunction interface, thus resulting in Z-scheme heterojunction formation. Various characterization methods such as XPS, UV-DRS, electrochemical impedance spectroscopy, photoluminescence, FESEM, TEM, and photocurrent response measurements were explored to explain the optical and electrochemical properties of the semiconductor heterojunction. The PEC degradation process was optimized, demonstrating a degradation efficiency removal of 80% for 5 mg/L sulfamethoxazole in water, with a TOC removal of 45.5%. A Z-scheme heterojunction formation mechanism was proposed to explain the enhanced photoelectrocatalytic activity of the photoanode. This work generally contributes to the development of efficient and sustainable photoanodes for environmental remediation.

## 1 Introduction

In recent years, the degradation of organic pollutants in water via the photoelectrocatalytic oxidation process (PEC) has emerged as a promising approach to tackle environmental threats, such as water pollution. PEC involves the integration of electrochemistry into a photocatalytic process by applying an external potential to the photoanode, thus enhancing the efficiency of pollutant degradation.^1−3^ Semiconductor photocatalysts have attracted significant attention for their potential in the PEC for pollutant degradation because of their ability to harness solar energy and generate reactive oxygen species (ROS).^4^ In the PEC, the semiconductor absorbs photons from light irradiation to generate pairs of electrons and holes. These photoexcited charge carriers can participate in oxidation and reduction reactions at the surface of the photocatalyst to achieve the degradation of organic pollutants into harmless byproducts.^5^

Effective PEC performance requires the development of photoanodes based on a comprehensive understanding of charge transfer mechanisms, surface reactivity, degradation pathways, and semiconductor properties such as conductivity and visible light absorbance.^6^ In addition, PEC water oxidation is a critical step for developing sustainable PEC systems. Water oxidation involves the generation of oxygen molecules from water, driven by photogenerated holes. This reaction plays a key role in overall PEC processes and has similar mechanisms to the degradation of pollutants, such as efficient mobility of electrons, charge separation, and photocatalyst stability.^7^ Recent breakthroughs in PEC processes have focused on the development of advanced photoanode materials with optimized band structures and enhanced photoelectrocatalytic activity through morphology tuning and multilayered structure,^8^ as well as investigating the role of substrates on the stability and efficiency of PEC systems.^9^ Notably, heterojunction photoanodes have shown significant promise due to their ability to improve charge separation and reduce electron–hole recombination, leading to an increased photocurrent and higher oxidation efficiency.

Bismuth-based compounds, particularly bismuth oxychalcogenides, have emerged as promising semiconductors due to their unique electronic layered framework and visible light absorption properties.^10^ The Bi 6s and Bi 6p orbitals of bismuth-based compounds influence the valence band and conduction band position, leading to a reduced band gap.^11^ A member of this group is bismuth oxysulfide (Bi_2_O_2_S), which stands out due to its low band gap that allows for visible light absorption, and its unique electronic structure, which contributes to enhanced charge transfer and chemical stability.^12^ Bi_2_O_2_S has a layered structure, in which two layers of [Bi_2_O_2_]^2+^ are separated by a column of S^2–^ anions.^13^ Being a layered semiconductor, it possesses a built-in electric field and has the ability to generate charge carriers in a nonequilibrium pattern between [Bi_2_O_2_]^2+^ and S^2–^ under light irradiation.^14^ These unique properties make Bi_2_O_2_S a promising photocatalyst for PEC oxidation. In contrast, nickel titanate (NiTiO_3_), a low-cost environmentally friendly perovskite, has proven to be a promising candidate for photoelectrocatalytic processes due to its diverse physiochemical and light absorption properties as well as its narrow band gap that ranges between 2.2 and 3.2 eV.^15,16^ However, a single semiconductor is known to experience a fast recombination rate of photogenerated charge carriers, leading to an overall low degradation efficiency.^17^ A major factor that is considered in the formation of semiconductor heterojunctions is the band alignment between the two semiconductors, that is, the position of the valence band and conduction band corresponding to the band gap energy.^18^ Several band alignments, such as types I–III, have been proposed for the semiconductor heterojunctions.^17^ However, the most common in PEC processes is the type II band alignment, which has led to the existence of Z- and S-scheme heterojunctions.^19^ In addition to the unique properties of Bi_2_O_2_S and NiTiO_3_, the type II band alignment of their band positions can give rise to a Z-scheme, which can facilitate better separation of photogenerated charge carriers. For instance, Guo et al. reported improved charge transfer and separation efficiency of a Z-scheme semiconductor heterojunction of NiTiO_3_ and g-C_3_N_4_, which improved the overall degradation efficiency when compared with that of the pristine semiconductors.^20^

Moreover, heterojunction-based systems with semiconductors of different energy levels have gained prominence because they mitigate fast recombination rates, thus leading to enhanced charge separation and improved photoelectrocatalytic performance.^21^ In addition, heterojunction photocatalysts with interfacial bonds have demonstrated considerable redox potential because of the strong interaction between the two semiconductors. The occurrence of distinct band alignment and internal electric field can lead to efficient electron migration and, consequently, charge separation. For instance, Li et al.^22^ fabricated a Mn_0.5_Cd_0.5_S/BiOBr S-scheme photocatalyst and confirmed that the formation of a covalent Bi–S bond at the heterojunction interface as well as the internal electric field between Mn_0.5_Cd_0.5_S and BiOBr enhanced the light response, facilitated the separation and segregation of photoexcited charge carriers, and maximized the redox potential, thereby significantly boosting the photocatalytic performance of Mn_0.5_Cd_0.5_S/BiOBr. Also, Ai et al.^23^ constructed an S-scheme ZnO/In_2_S_3_ heterojunction and confirmed that the formation of an S–O covalent bond at the heterojunction interface promoted efficient charge separation and enhanced the photocatalytic properties.

Several types of semiconductor heterojunction formation, such as p–n,^24,25^ n–n,^26,27^ Z-scheme,^28,29^ and S-scheme,^30^ among others, have been reported and proven noteworthy due to their unique mechanisms and performances. Owing to energy level differences and band positions, the combination of Bi_2_O_2_S and NiTiO_3_ nanoparticles will form a heterojunction with better charge transfer and separation, suppressed rate of recombination, as well as enhanced degradation efficiency. Among several organic pollutants, sulfamethoxazole, a commonly used antibiotic, is frequently detected in aquatic environments, particularly in surface water and wastewater treatment plants. The persistence of sulfamethoxazole in water bodies arises primarily from incomplete metabolism and excretion by humans and animals as well as improper disposal of unused medications. As an organic pollutant, sulfamethoxazole poses environmental and health concerns due to its persistence, bioaccumulation potential, and adverse effects on aquatic ecosystems and humans.^31,32^ Therefore, in this study, we report the in situ synthesis, fabrication, and characterization of Z-scheme Bi_2_O_2_S/NiTiO_3_ for the photoelectrocatalytic degradation of sulfamethoxazole in water. The in situ synthesis of Bi_2_O_2_S/NiTiO_3_ led to the formation of an S–O bond (between the sulfur atom of Bi_2_O_2_S and oxygen atoms of NiTiO_3_), at the heterojunction interface, thereby causing a strong interaction and easy migration of electrons. Also, the band alignment of Bi_2_O_2_S/NiTiO_3_ gives rise to a Z-scheme heterojunction with an internal electric field, which serves as a driving force for charge separation and suppresses the recombination rate of photoexcited charges. X-ray photoelectron spectroscopy (XPS) and photoluminescence were used to investigate the electronic interactions between Bi_2_O_2_S and NiTiO_3_. We examined the optical and electrochemical characteristics of the photoanodes, conducted photodegradation experiments of SMX, and investigated the radical generation, degradation pathways, photoanode stability, and charge pathway involved in the formation of the Z-scheme heterojunction. This study aims to contribute to the development of efficient photo(electro)catalysts with improved interfaces for organic pollutant degradation through a unique combination of semiconductors with suitable band gaps to address pressing environmental challenges. Moreover, the insights gained from this research could pave the way for the fabrication, design, and optimization of heterojunction-based photocatalysts.

## 2 Experimental Section

### 2.1 Materials

The materials and chemicals used in this work are listed in the Supporting Information.

### 2.2 Synthesis of NiTiO_3_

The NiTiO_3_ photocatalyst was synthesized using the method reported by Ojo et al.^33^ Ni(CH_3_CO_2_)_2_·4H_2_O (0.02 M) was dispersed in a beaker containing a mixture of acetic acid (CH_3_COOH) and ethylene glycol (C_2_H_6_O_2_). The mixture was sonicated for 15 min, and then 0.032 M tetra-*n*-butyl ortho-titanate ((CH_3_CH_2_CH_2_CH_2_O)_4_Ti) was added. The mixture was then stirred for 1 h, followed by heating in an oven for 1 h at 120 °C. The obtained product was calcined in a furnace at 800 °C for 3 h to obtain a NiTiO_3_ yellowish powder.

### 2.3 Synthesis of Bi_2_O_2_S

Bi_2_O_2_S nanoparticles were synthesized using a hydrothermal method, as reported in our previous work.^34^ In this process, 1.9403 g of bismuth nitrate pentahydrate (Bi(NO_3_)_3_·5H_2_O) and 0.1522 g of thiourea (CH_4_N_2_S) were dissolved in 50 mL of deionized water, and the mixture was sonicated for 20 min, after which 12 g of lithium monohydrate (LiOH·H_2_O) was added. The mixture was sonicated for 50 min to obtain a uniformly reddish solution. This solution was then transferred to an 80 mL Teflon-lined hydrothermal autoclave and heated at 200 °C for 72 h. After cooling the autoclave, the obtained product was washed multiple times with deionized water and absolute ethanol via centrifugation and dried at 80 °C for 8 h.

### 2.4 Synthesis of Bi_2_O_2_S/NiTiO_3_

The Bi_2_O_2_S/NiTiO_3_ nanocomposite was synthesized via an in situ hydrothermal method, in which three different mole ratios (5, 15, and 25% mole ratios of NiTiO_3_ to Bi_2_O_2_S) of the previously synthesized NiTiO_3_ were dispersed in deionized water and sonicated for 15 min, followed by the synthesis of Bi_2_O_2_S, as reported in Section 2.3.

### 2.5 Fabrication of the Photoanode

A Bi_2_O_2_S/NiTiO_3_ photoanode was fabricated via a drop-coating method using fluorine-doped tin oxide (FTO) glass as the conducting substrate. FTO was sonicated for 5 min with acetone, rinsed with deionized water, and dried at 60 °C for 2 h. Next, a slurry mixture consisting of the synthesized Bi_2_O_2_S/NiTiO_3_ nanocomposite (50 mg) and poly(vinylidene fluoride) (5 mg) dispersed in *N*-methyl-2-pyrrolidone (90 μL) was carefully drop-coated onto FTO glass to achieve a uniform film and dried at 80 °C for 2 h. The geometric area of the photoanode Bi_2_O_2_S/NiTiO_3_ was 1.7 × 1.7 cm^2^. The same procedure was repeated to fabricate FTO/NiTiO_3_ and FTO/Bi_2_O_2_S photoanodes.

Details of the instrument characterization, including X-ray diffraction, X-ray photoelectron spectroscopy, field-emission scanning electron microscopy, transmission electron microscopy, UV–vis diffuse reflectance spectrophotometry, photoluminescence spectroscopy, ζ-potential analyzer, total organic carbon analysis, contact angle measurement, time-resolved photoluminescence, photo(electro)chemical measurements, mass spectrometry, and photoelectrochemical degradation setup and procedure are provided in the Supporting Information.

## 3 Results and Discussion

### 3.1 Structural, Morphological, Elemental, and Contact Angle Studies

**Figure 1:**
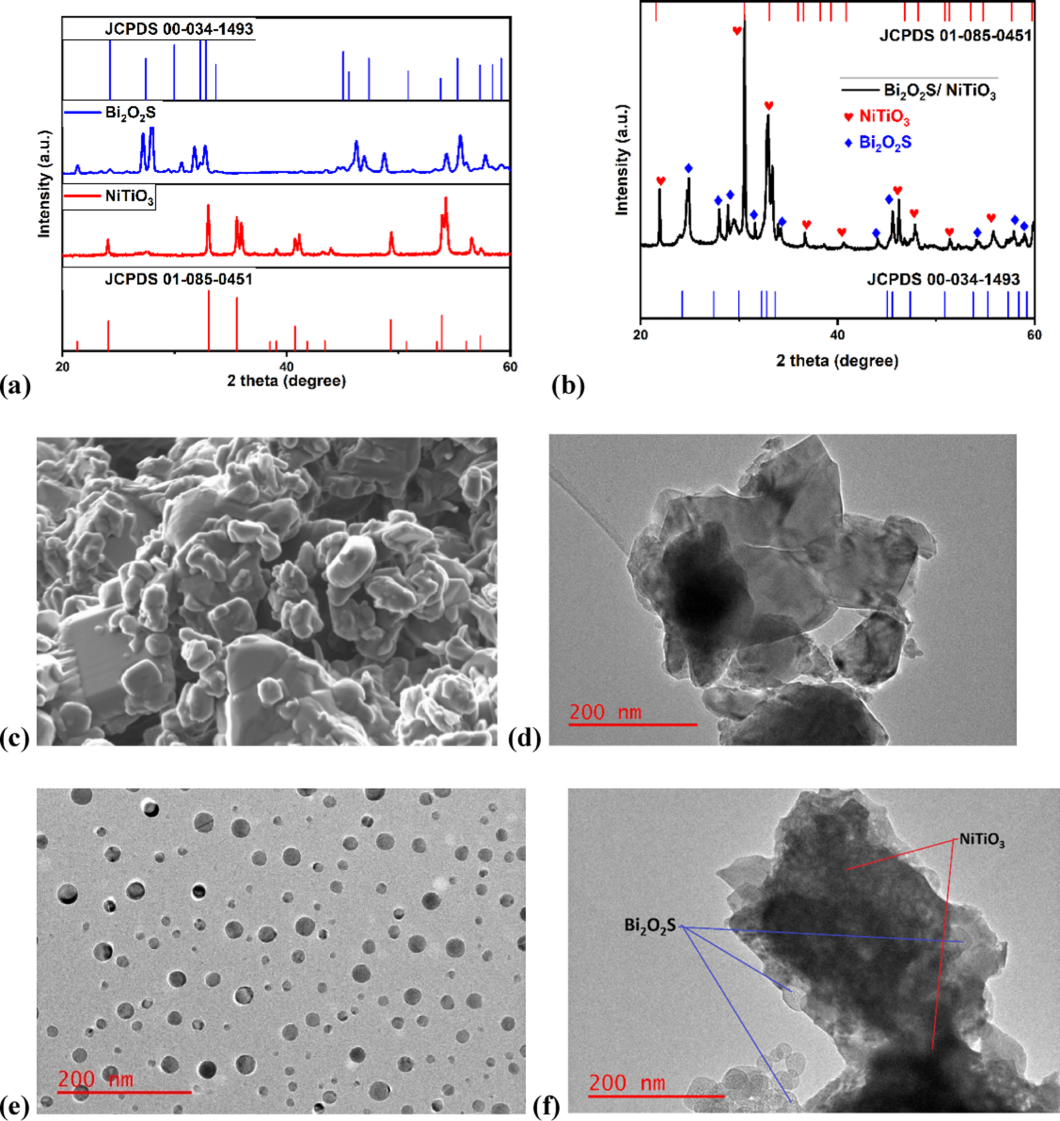
XRD patterns of (a) NiTiO_3_ and (b) Bi_2_O_2_S/NiTiO_3._ (c) FESEM micrograph of Bi_2_O_2_S/NiTiO_3_, and TEM micrographs Bi_2_O_2_S of (d) NiTiO_3_, (e) Bi_2_O_2_S, and (f) Bi_2_O_2_S/NiTiO_3_.

X-ray diffraction (XRD) analysis was used to investigate the successful synthesis of semiconductors. Figure 1a shows the diffraction patterns of the synthesized Bi_2_O_2_S and NiTiO_3_. Bi_2_O_2_S has its main peaks at 27.6, 30.4, 32.1, 33.1, 46.3, 48.7, 54.2, 55.5, and 57.8°, which are indexed to the *hkl* (120), (040), (130), (111), (060), (002), (151), (112), and (161) planes of the Bi_2_O_2_S orthorhombic phase with JCPDS #00-034-1493.^34^ NiTiO_3_ has the characteristics peak at 2θ = 24.2° (012), 32.9° (104), 39.0° (006), 41.08° (021), 43.92° (202), 49.3° (024), 54.2° (116̅), and 56.5° (121̅), which can be attributed to the NiTiO_3_ rhombohedral phase (JCPDS #01-085-0451). All of these major peaks were observed in the diffraction peaks of Bi_2_O_2_S/NiTiO_3_ (Figure 1b), with a slight shift in the 2θ values of Bi_2_O_2_S. The shift to a lower 2θ observed in the *hkl* plane of (120), and to a higher 2θ observed in (040) and (130) was due to the in situ preparation of the heterojunction. These strain-induced defects can affect the optical and electrical properties, thus influencing the overall degradation efficiency of a semiconductor.^35^

The FESEM micrograph of NiTiO_3_ (Figure S1) shows a plate-like structure covered with numerous smaller clustered particles, indicating a rough textured surface. In contrast, the FESEM micrograph of Bi_2_O_2_S (Figure S2) shows a cluster of small unevenly shaped crystals. Some particles appear to have a layered structure, which suggests a combination of different pore geometries. The FESEM micrograph of Bi_2_O_2_S/NiTiO_3_ (Figure 1c) shows an overlap of both Bi_2_O_2_S and NiTiO_3_ particles due to the preparation method, indicating that the semiconductors are closely intertwined with each other. In addition, the TEM micrograph of NiTiO_3_ (Figure 1d) confirms the plate-like structure, while the TEM micrograph in Figure 1e shows a well-dispersed spherical morphology. Furthermore, the TEM micrograph of Bi_2_O_2_S/NiTiO_3_ (Figure 1f) shows a combination of NiTiO_3_ and Bi_2_O_2_S particles. However, the monodispersed Bi_2_O_2_S particles were observed to be in small clusters around and on the distorted shape of NiTiO_3_. This could explain the shift observed in the 2θ values of Bi_2_O_2_S/NiTiO_3_ due to the slight change in the morphology when compared to pristine Bi_2_O_2_S and NiTiO_3_. Thus, this confirmed the formation of a heterojunction.

**Figure 2:**
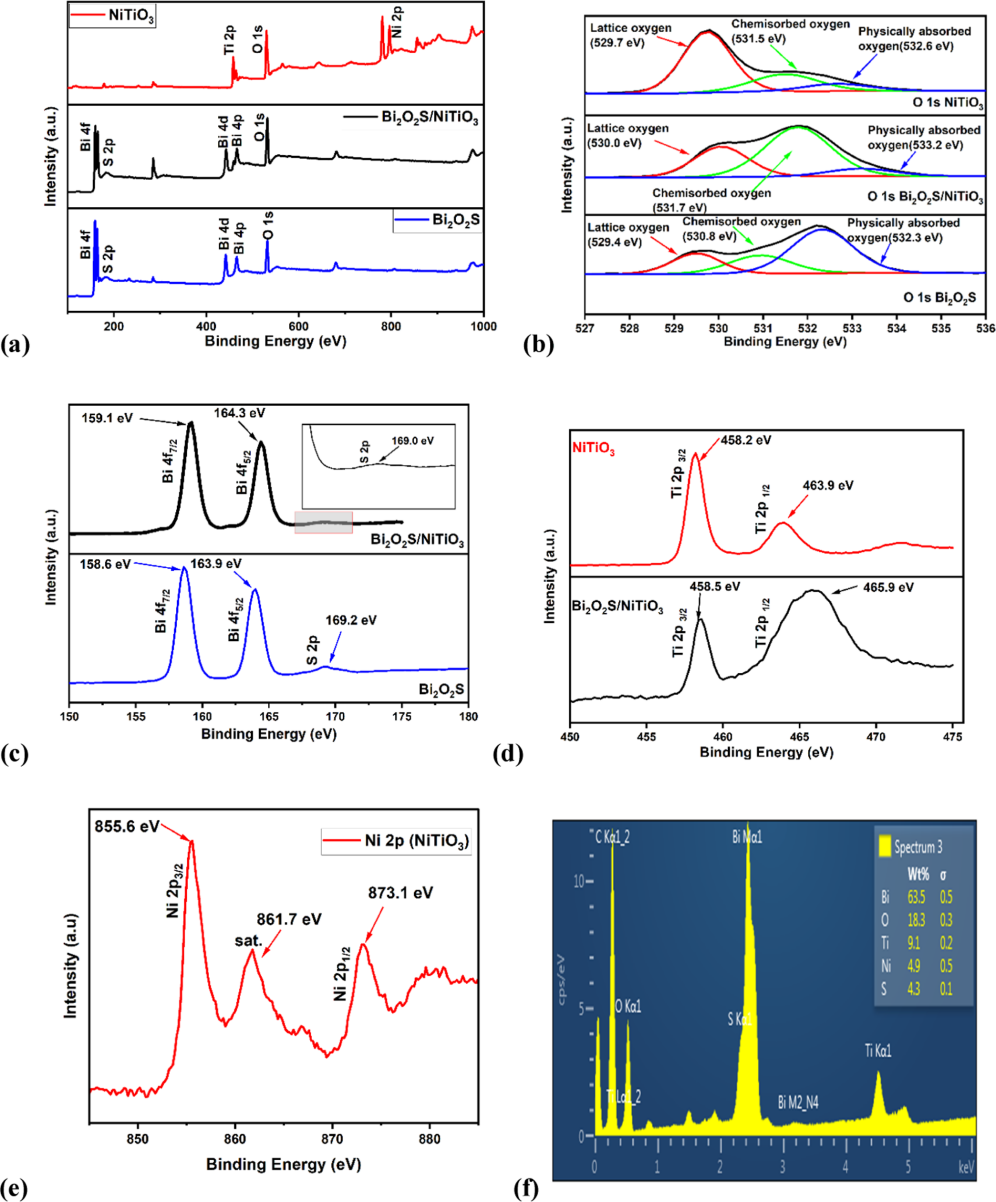
(a) XPS survey scan of Bi_2_O_2_S/NiTiO_3_. XPS spectra of O 1s of Bi_2_O_2_S, NiTiO_3_, and Bi_2_O_2_S/NiTiO_3_ (b), Bi 4f and S 2p of Bi_2_O_2_S and Bi_2_O_2_S/NiTiO_3_ (c), Ti 2p of NiTiO_3_, and Bi_2_O_2_S/NiTiO_3_ (d), Ni 2p of NiTiO_3_ (e), and SEM/EDX of Bi_2_O_2_S/NiTiO_3_ (f) FESEM-EDX spectra of Bi_2_O_2_S/NiTiO_3_.

The valence states and chemical compositions of NiTiO_3_, Bi_2_O_2_S, and Bi_2_O_2_S/NiTiO_3_ were determined by XPS. Figure 2a shows the survey scans of NiTiO_3_, Bi_2_O_2_S, and Bi_2_O_2_S/NiTiO_3_, revealing the presence of their corresponding elements nickel, titanium, bismuth, sulfur, and oxygen. Figure 2b shows the O 1s spectral peaks at 529.7 531.5, and 532.6 eV for NiTiO_3_; 529.4, 530.8, and 532.3 eV for Bi_2_O_2_S, which corresponds to the metal–oxygen bonds, chemisorbed oxygen, and physically absorbed oxygen, respectively, in both Bi_2_O_2_S and NiTiO_3_.^36^ In comparison, the binding energies of the metal–oxygen bond, chemisorbed oxygen, and physically absorbed oxygen increased to 530.0, 531.7 and 533.2 eV, respectively, for the O 1s of Bi_2_O_2_S/NiTiO_3_.This observed increase in the binding energy could be attributed to the interactions between Bi_2_O_2_S and NiTiO_3_ in the Bi_2_O_2_S/NiTiO_3_ heterojunction. In Figure 2c, the peaks observed at 158.6, 163.9, and 169.2 eV for Bi_2_O_2_S correspond to Bi 4f_7/2_, Bi 4f_5/2_, and S 2p orbitals, which were observed to shift to 159.1, 164.3, and 169.0 eV in Bi_2_O_2_S/NiTiO_3._ The Ti 2p_3/2_ and 2p_1/2_ orbital spectra for NiTiO_3_ and Bi_2_O_2_S/NiTiO_3_ (Figure 2d) were observed at 458.2 and 463.9 eV and 458.5 and 465.9 eV, respectively. Furthermore, the XPS spectra displayed in Figure 2e show peaks of Ni 2p_3/2_ and 2p_1/2_ of NiTiO_3_ at 855.6 and 873.1 eV, respectively, and a satellite peak at 861.7 eV, which is indicative of the Ni^2+^ oxidation state. However, Ni 2p was not detected in Bi_2_O_2_S/NiTiO_3_ because XPS is a surface-sensitive instrument that can only analyze the elemental composition of the top 10 nm of the sample surface. From the preparation method of Bi_2_O_2_S/NiTiO_3_, Bi_2_O_2_S nanoparticles were synthesized on NiTiO_3_ nanoparticles. Therefore, factors such as surface coverage of Bi_2_O_2_S, charge redistribution, and change in the composition can reduce the presence of nickel in the outermost layer, thus making it undetected by XPS.^37^ However, to confirm the presence of nickel in the Bi_2_O_2_S/NiTiO_3_ heterojunction, scanning electron microscopy with energy-dispersive X-ray spectroscopy (SEM/EDX) was used. As displayed in Figure 2f, the presence of bismuth, nickel, titanium, sulfur, and oxygen in Bi_2_O_2_S/NiTiO_3_ was confirmed. In summary, the findings from the XPS analysis revealed that the binding energies of Bi 4f, Ti 2p, and O 1s shifted to a higher binding energy, while the S 2p orbitals shifted to a lower binding energy when compared with the respective orbitals in Bi_2_O_2_S and NiTiO_3_. These results suggest that the in situ synthesis of Bi_2_O_2_S on NiTiO_3_ to form a heterojunction leads to the redistribution of electrons in NiTiO_3_. Moreover, the shift to lower binding energy observed in the sulfur orbital suggests an increase in the electron density (electron accumulation), thus suggesting the presence of S–O bonds at the interface of the Bi_2_O_2_S/NiTiO_3_ heterojunction, leading to the detection of an additional S 2s peak in Bi_2_O_2_S/NiTiO_3_ (Figure S3).

The FTIR spectra of Bi_2_O_2_S, NiTiO_3_, and Bi_2_O_2_S/NiTiO_3_ (Figure S4) show the functional groups present in the semiconductor. The absorption bands of Bi_2_O_2_S at ≈488, 866, and 1411 cm^–1^ correspond to the Bi–O bending vibration, Bi–O stretching vibration, and S–O bending vibration, respectively.^34^ The band position of NiTiO_3_ at ≈602 cm^–1^ is attributed to Ni–O bending vibrations, typically of the metal–oxygen bending mode, while the absorption bands at 1428, 1643, and 3442 cm^–1^ correspond to the Ti–O stretching vibrations and O–H stretching vibration due to the presence of absorbed water molecules on the surface of NiTiO_3_.^38,39^ In the spectra of Bi_2_O_2_S/NiTiO_3_, in addition to the corresponding absorption bands (417, 612, 1638, and 3447 cm^–1^) observed in pristine Bi_2_O_2_S and NiTiO_3_, an additional peak appears at the band position 1131 cm^–1^, which corresponds to the S–O stretching vibration, respectively, due to heterojunction formation.^40^ Thus, corroborating the XPS analysis suggests the presence of an S–O bond at the interface of the Bi_2_O_2_S/NiTiO_3_ heterojunction.

Contact angle measurements were performed to investigate the surface wettability of the photoanode (Figure S5). Low contact angles of less than 90° correspond to high wettability, while high contact angles of greater than 90° suggest low wettability. Moreover, this method is used to investigate the hydrophilicity of the photoanode. How a photocatalyst reacts with water is also an important factor that affects its degradation efficiency. Average contact angles of 101.7, 124.3, and 92.2° were observed for Bi_2_O_2_S, NiTiO_3,_ and Bi_2_O_2_S/NiTiO_3_, respectively, indicating that the formation of the heterojunction also increased the hydrophilicity of the photoanode.

### 3.2 Optical and Photo(electro)chemical Properties

**Figure 3:**
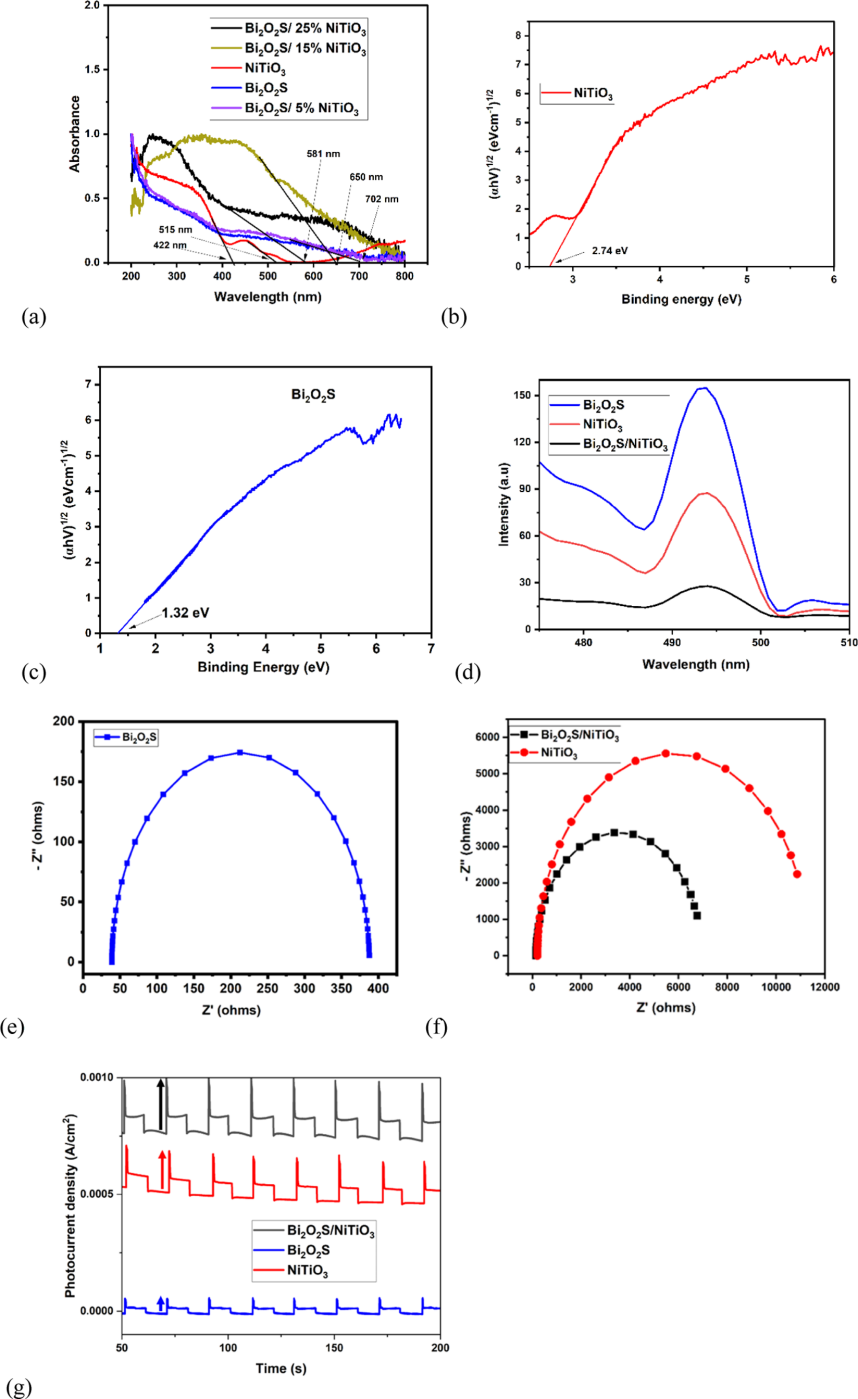
(a) UV/vis DRS spectra of Bi_2_O_2_S, NiTiO_3_, and Bi_2_O_2_S/NiTiO_3_. Tauc plots of (b) NiTiO_3_ and (c) Bi_2_O_2_S. (d) Steady-state Pl spectra. EIS spectra of (e) Bi_2_O_2_S, (f) NiTiO_3_, and Bi_2_O_2_S/NiTiO_3_ (5 mM [Fe(CN)6]^3/4–^ in 0.1 M KCl, +0.25 V, 100 kHz to 0.1 Hz). (g) Transient photocurrent response plot of Bi_2_O_2_S, NiTiO_3_, and Bi_2_O_2_S/NiTiO_3_ (0.1 M Na_2_SO_4_).

Pristine Bi_2_O_2_S shows strong absorption of light in the visible light region with an absorption edge at 710 nm (Figure 3a). NiTiO_3_ shows two absorption peaks at 422 and 515 nm, which are a result of the crystal field splitting effect arising from the Ni^2^–Ti^4+^ charge transfer band.^41^ However, the Bi_2_O_2_S/5% NiTiO_3_, Bi_2_O_2_S/15% NiTiO_3_, and Bi_2_O_2_S/25% NiTiO_3_ heterojunction semiconductors gave absorption wavelengths of absorbed light in a wider range in the visible region of the electromagnetic spectrum with an absorption edge of 511 nm. This improved light absorption results from the formation of a heterojunction, which can lead to the enhanced photoelectrochemical activity of Bi_2_O_2_S/NiTiO_3_.

The band gap energies of Bi_2_O_2_S and NiTiO_3_ were extrapolated using the Tauc plot (eq 1)
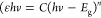
1where hv is the energy of the incident photon, ε is the coefficient of the molar extinction, *C* is a constant, in which the type of transition determines the value of *n*, and *E*_g_ is the band gap energy,

Based on the indirect allowed transition, the band energies for NiTiO_3_ (Figure 3b) and Bi_2_O_2_S (Figure 3c) are 2.74 and 1.32 eV, respectively. The heterojunction formation between NiTiO_3_ and Bi_2_O_2_S resulted in a band gap of 1.8 eV for Bi_2_O_2_S/NiTiO_3_ (Figure S6), thus suggesting the photoanode to be a good solar (visible) light-harvesting electrode. The band gap energy obtained can be used to determine the conduction band (CB) and valence band (VB) energies theoretically based on the electron affinity (eqs 2 and 3).
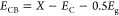
2
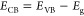
3where *X* is the absolute electronegativity, *E*_g_ is the band gap energy, *E*_CB_ is the conduction band energy, and *E*_C_ is the free electron energy on the hydrogen scale, which is usually equal to 4.5 eV.

The absolute electronegativities of Bi_2_O_2_S and NiTiO_3_ were determined to be 4.81^34^ and 5.6 eV,^42^ respectively. The calculated conduction bands of Bi_2_O_2_S and NiTiO_3_ are −0.34 and −0.27 eV, respectively. The *E*_VB_ values of Bi_2_O_2_S and NiTiO_3_ are 0.94 and 2.47 eV, respectively. In agreement with the theoretical calculation of the conduction bands of Bi_2_O_2_S and NiTiO_3_, the flat band potentials (*V*_f_) of Bi_2_O_2_S (Figure S7) and NiTiO_3_ (Figure S8) deduced from the Mott–Schottky plot are −0.33 and −0.28 V vs Ag/AgCl electrode, respectively. Using the formula *E*_(NHE)_ = *E*_(Ag/AgCl)_ + 0.194, the *V*_f_ values corresponding to the normal hydrogen electrode are −0.13 and −0.08 V for Bi_2_O_2_S and NiTiO_3_ photoanodes, respectively. Generally, for an n-type semiconductor, the conduction band is more negative than the flat band by approximately 0.1–0.2 V. Therefore, it is expected that the conduction bands of Bi_2_O_2_S and NiTiO_3_ are approximately close to *V*_f_, which was observed in this case, confirming the calculated conduction bands of Bi_2_O_2_S and NiTiO_3_ as −0.34 and −0.27 eV, respectively

Furthermore, based on the XPS valence spectra of Bi_2_O_2_S (Figure S9) and NiTiO_3_ (Figure S10), the valence band maxima (VBM) of Bi_2_O_2_S and NiTiO_3_ were deduced to be 0.54 and 2.04 eV, respectively. The discrepancy between the *E*_VB_ (NHE) and *E*_VB_ (XPS) is due to the fact that the binding energy obtained in *E*_VB_ (XPS) is with respect to the Fermi level and not the vacuum level.^43^ However, eq 4 can be used to confirm the calculated *E*_VB_ (NHE)
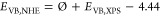
4where ⌀ is the work function of the XPS instrument, and is given as 4.8 eV. Using this formula, the expected *E*_VB_ values (NHE) for Bi_2_O_2_S and NiTiO_3_ are 0.90 and 2.44 eV, respectively. These values are in agreement with the calculated *E*_VB_ (NHE), with a difference of less than 0.05 eV.

In addition, the slopes of the Mott–Schottky curves of Bi_2_O_2_S (2.01 × 10^9^) and NiTiO_3_ (5.72 × 10^11^) were used to estimate the concentration of the major charge carrier using eq 5.
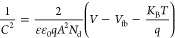
5where *C* is the capacitance, ε is the dielectric constant, ε_0_ is the permittivity of free space, *q* is the charge of the electrons, *A* is the surface area, *N*_d_ is the concentration of the major charge carrier, *V* is the applied voltage, *V*_fb_ is the flat band potential, *K*_B_ is the Boltzmann constant, and *T* is the absolute temperature.^44^

Equation 5 shows that the slope is inversely proportional to the concentration of the major charge carriers (approximately equal to the electron density). A higher electron density in n-type semiconductors indicates that the Fermi level is closer to the conduction band.^45^ Hence, with respect to the conduction bands of the semiconductors, the Fermi level of Bi_2_O_2_S is higher than that of NiTiO_3._

Photoluminescence spectroscopy was used to determine whether the rate of the recombination of photogenerated holes and electrons was suppressed after the formation of the heterojunction. A strong photoluminescence intensity indicates a high recombination rate of the photogenerated charge carriers, which can influence the photocatalytic efficiency of semiconductors, while a reduction in photoluminescence intensity is evidence for a lower rate of recombination.^46^ The photoluminescence intensity of the Bi_2_O_2_S/NiTiO_3_ heterojunction is lower than that of pristine Bi_2_O_2_S and NiTiO_3_ (Figure 3d), which implies that the recombination rate of the photogenerated holes and electrons was suppressed due to the heterojunction formation between Bi_2_O_2_S and NiTiO_3_ nanoparticles, suggesting effective charge separation.

The time-resolved photoluminescence decay spectra were collected to further understand the charge carrier dynamics. Using the single exponential decay fitting (eq 6), as shown in Figure S11, the average lifetimes of 3.05, 2.95, and 1.07 ns were obtained for Bi_2_O_2_S, NiTiO_3_, and Bi_2_O_2_S/NiTiO_3,_ respectively. The reduction in the lifetime of the Bi_2_O_2_S/NiTiO_3_ heterojunction suggests enhanced charge separation and improved interfacial charge transfer. This implies that photogenerated charge carriers migrate across the interface of the heterojunction rather than recombining within the same semiconductor.^47^ This further explains the suppressed rate of recombination observed in Bi_2_O_2_S/NiTiO_3_ heterojunction
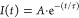
6where *I*(*t*) is the intensity, *A* is the amplitude at time *t*, and τ is the decay lifetime.

Charge transfer resistance (*R*_ct_) values from Nyquist plots of electrochemical impedance spectroscopy with the equivalent circuit model (Figure S12) were 348.62 Ω (Figure 3e), 11,131 Ω (Figure 3f), and 6785.2 Ω (Figure 3f) for Bi_2_O_2_S, NiTiO_3_, and Bi_2_O_2_S/NiTiO_3_ photoanodes, respectively. The lowest *R*_ct_ of 348.62 Ω shows that the Bi_2_O_2_S photoanode is more conductive; thus, it contributes to the overall conductivity of the Bi_2_O_2_S/NiTiO_3_ photoanode heterojunction.

In addition, zeta analysis was used to investigate the surface charge and average ζ-potential of the Bi_2_O_2_S, NiTiO_3_, and Bi_2_O_2_S/NiTiO_3_ nanoparticles. External factors, such as surface charge, have been proven to affect the efficiency of a photocatalyst.^48^ Moreover, a large negative or positive ζ-potential (≥30 mV) indicates the potential stability of the semiconductor. As shown in Table 1, the ζ-potential values of the semiconductors indicate that the Bi_2_O_2_S/NiTiO_3_ nanoparticles are more stable than the pristine ones. The conductivity values in Table 1 indicate that Bi_2_O_2_S is the most electrically conductive. The presence of Bi_2_O_2_S in the Bi_2_O_2_S/NiTiO_3_ heterojunction improved the conductivity of NiTiO_3_, which is in agreement with the *R*_ct_ values obtained from impedance spectroscopy. In addition, the point of zero charge (PZC) of Bi_2_O_2_S/NiTiO_3_ in an aqueous solution was found to be 2.7 (Figure S13), which implies that at pH greater than 2.7, the surface of Bi_2_O_2_S/NiTiO_3_ is negatively charged, while at pH less than 2.7, the surface of Bi_2_O_2_S/NiTiO_3_ is positively charged, and at pH approximately equal or closer to 2.7, Bi_2_O_2_S/NiTiO_3_ is neutral.

**Table 1:** ζ-Potential and Conductivity Values for Bi_2_O_2_S, NiTiO_3_, and Bi_2_O_2_S/NiTiO_3_

semiconductor	surface charge and average ζ-potential (mV)	electrical conductivity (mS/cm)
Bi_2_O_2_S	–49.6	0.021
NiTiO_3_	+18.2	0.002
Bi_2_O_2_S/NiTiO_3_	–51.0	0.009

The photocurrent responses of the electrodes were investigated; as shown in Figure 3g, the Bi_2_O_2_S/NiTiO_3_ photoanode depicts a higher photocurrent density of 0.230 mA/cm^2^ while the pristine Bi_2_O_2_S and NiTiO_3_ have photocurrent densities of 0.0522 and 0.1802 mA/cm^2^, respectively. The spike observed in the photocurrent response is also known as decay, which implies that there is an accumulation of the minor charge carrier (photogenerated holes) on the surface of the photoanode, which leads to band bending. Moreover, a higher degree of band bending leads to increased charge separation and a decreased recombination rate for photogenerated holes and electrons,^49^ as observed in the heterojunction. Hence, the formation of a Bi_2_O_2_S/NiTiO_3_ heterojunction provides the advantage of a better photocatalyst with a lower recombination rate of the photoexcited charge carriers.

### 3.3 Photoelectrochemical Degradation of Sulfamethoxazole

**Figure 4:**
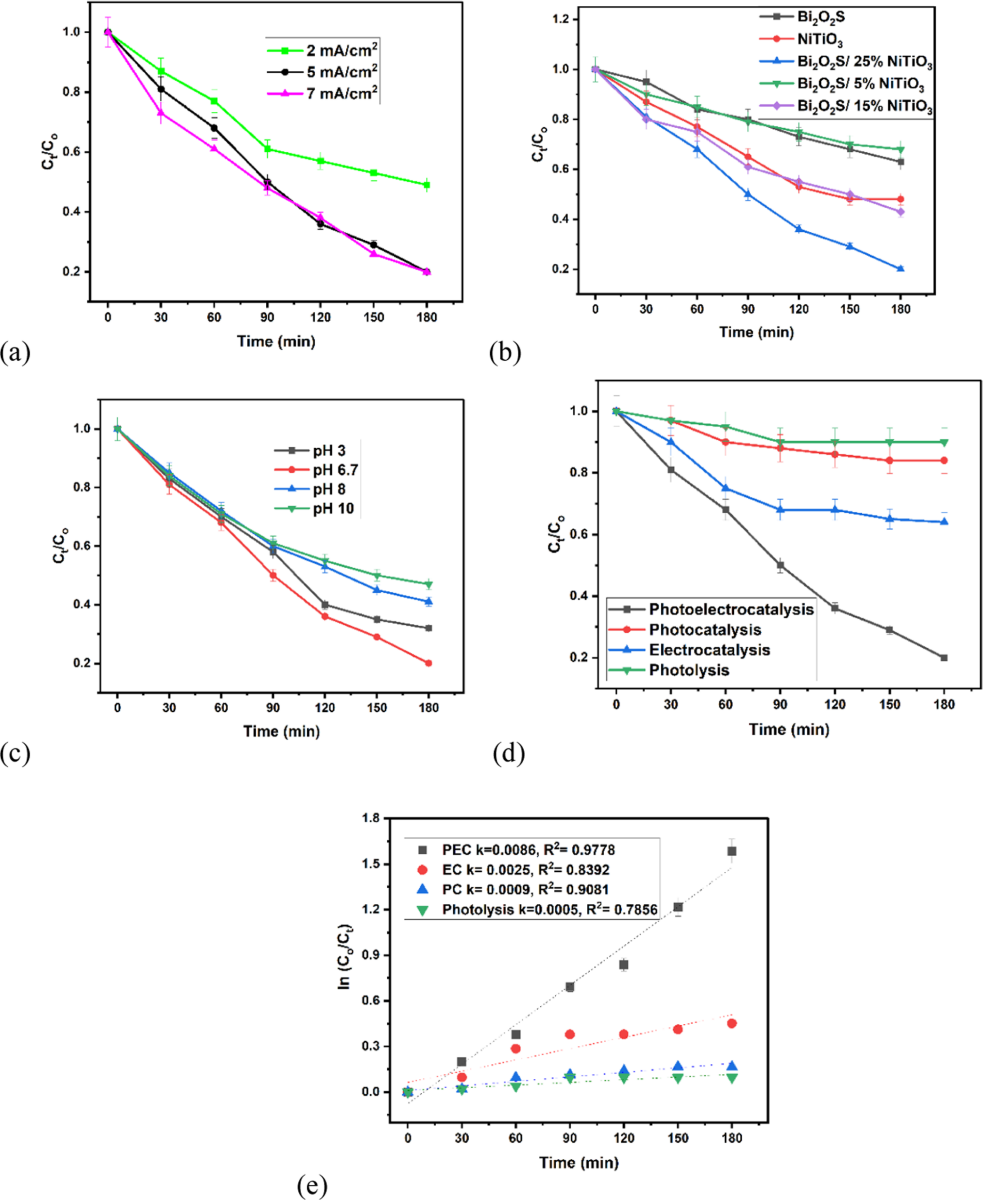
Plot of PEC degradation efficiency showing the (a) effect of current density, (b) effect of heterojunction, (c) effect of pH, (d) synergistic effect, and (e) pseudo-first-order kinetic studies (experimental conditions: sulfamethoxazole concentration = 5 mg/L in 0.1 M Na_2_SO_4_ and current density = 5 mA/cm^2^).

Parameters, such as the applied current or voltage, play crucial roles in a photoelectrochemical setup. In a two-electrode system with an external power source, current density is the amount of current generated relative to the surface area of the photoanode. It was observed that the PEC degradation of 5 mg/L sulfamethoxazole in 0.1 M Na_2_SO_4_ over the Bi_2_O_2_S/NiTiO_3_ photoanode in 180 min increased with an increase in the photocurrent density from 2 to 7 mA/cm^2^ (Figure 4a).

The Bi_2_O_2_S/NiTiO_3_ photoanode degradation efficiency was calculated by using eq 7:
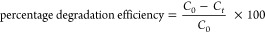
7where *C*_0_ is the concentration at time zero, and *C_t_* is the concentration at time *t*.

At 2 mA/cm^2^, the degradation efficiency was 51%, while at 5 and 7 mA/cm^2^, the efficiency was 80%. Hence, a current density of 5 mA/cm^2^ was taken as the optimal current density because the motivation was to use as low an energy consumption as possible. In addition, the effect of heterojunction formation on the photoelectrocatalytic properties of the semiconductors was investigated. As displayed in Figure 4b, the pristine Bi_2_O_2_S and NiTiO_3_ photoanodes showed degradation efficiencies of 37 and 52%, respectively, for the degradation of 5 mg/L sulfamethoxazole in 0.1 M Na_2_SO_4_ in 180 min, while the different mole ratios Bi_2_O_2_S/5% NiTiO_3_, Bi_2_O_2_S/15% NiTiO_3_, and Bi_2_O_2_S/25% NiTiO_3_ exhibited degradation efficiencies of 32, 57, and 80% respectively. Therefore, the Bi_2_O_2_S/25% NiTiO_3_ photoanode exhibited the highest degradation efficiency and was used as the optimal photocatalyst ratio.

The pH of the solution plays a significant role in determining the removal rate of sulfamethoxazole as it influences the surface charge and oxidation potential of the photoanode. Hence, the degradation efficiency of the Bi_2_O_2_S/NiTiO_3_ photoanode was investigated at different pH values. As shown in Figure 4c, the highest degradation efficiency of 80% was observed at pH 6.7, as opposed to pH 3 (68%), pH 8 (59%), and pH 10 (53%). Since the p*K*_a_ values of sulfamethoxazole are 1.8 and 5.7, at pH 6.7, sulfamethoxazole is partially ionized, and the surface of the Bi_2_O_2_S/NiTiO_3_ photoanode is negatively charged. This leads to better degradation efficiency.

In addition, the extent of sulfamethoxazole mineralization over the Bi_2_O_2_S/NiTiO_3_ photoanode was investigated via the total organic carbon (TOC) analysis, with a TOC removal percentage of 45.5% after 180 min (Figure S14). Thus, the heterojunction formation between Bi_2_O_2_S and NiTiO_3_ resulted in better photoelectrocatalytic properties, which could be attributed to better charge transfer, effective charge separation, and suppressed rate of recombination of photogenerated charge carriers.

Furthermore, the synergy of electrochemical oxidation and photocatalysis processes was investigated. As shown in Figure 4d, the photoelectrochemical oxidation process yields the highest degradation efficiency at the Bi_2_O_2_S/NiTiO_3_ photoanode. Lower degradation efficiencies of 16, 36, and 10% were obtained for the photocatalysis (PC), electrochemical (EC) oxidation, and photolysis (absence of the photocatalyst) processes, respectively. This suggests that combining photocatalysis and electrochemical oxidation can lead to a higher generation of reactive oxygen species. The data obtained was fitted to a pseudo-first-order reaction to examine the kinetics and synergic factors involved. The *k* values for the PEC, EC, PC, and photolysis processes (Figure 4e) are 0.0088, 0.0025, 0.0009, and 0.0005 min^–1^, respectively, indicating that the rate of degradation in the PEC process is much faster. Since PEC is a combination of PC and EC, a synergistic factor was calculated to support the markedly improved degradation percentage of PEC over EC and PC. From eq 8, a synergic factor of 2.59 was calculated, indicating a synergistic interaction between the PC and EC processes.
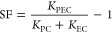
8

In addition, the data for the PEC degradation of SMX over the Bi_2_O_2_S/NiTiO_3_ photoanode was fitted to the pseudo-second-order model to further investigate the mechanism of the reaction between the photoanode and pollutant. As shown in Figure S15, a rate constant of 1.0205 min^–1^ was deduced from the slope, indicating a high reaction rate. However, an *R*^2^ value of 0.8225 was obtained, suggesting that the pseudo-second-order model does not perfectly fit the data, as compared to the *R*^2^ value of 0.9778 obtained for the pseudo-first-order model. Therefore, the reaction mechanism of the Bi_2_O_2_S/NiTiO_3_ photoanode with SMX involves physical interaction. This enables SMX to attach loosely on the photoanode, leading to fast degradation efficiency.^50^

### 3.4 Reusability and the Radical Trapping Test

**Figure 5:**
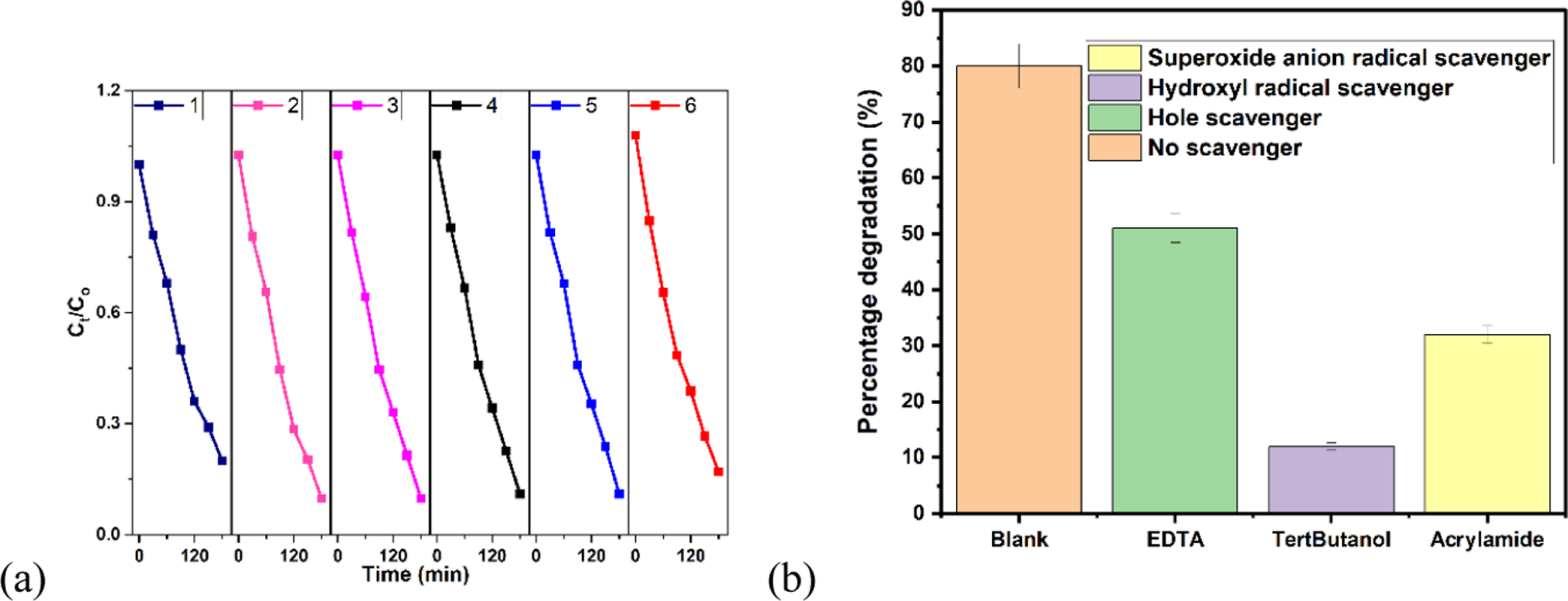
(a) Reusability study and (b) free radical determination study of the Bi_2_O_2_S/NiTiO_3_ photoanode (experimental conditions: sulfamethoxazole concentration = 5 mg/L in 0.1 M Na_2_SO_4_ and current density = 5 mA/cm^2^).

The reusability and stability of the Bi_2_O_2_S/NiTiO_3_ photoanode (Figure 5a) were investigated through a 6-cycle treatment of the degradation of sulfamethoxazole. A degradation efficiency of 80% was maintained until the fifth cycle, and a decrease in efficiency of 5% was observed during the sixth cycle. Hence, the Bi_2_O_2_S/NiTiO_3_ photoanode can be said to be stable and reusable until the sixth cycle of water treatment. In addition to the reusability and stability studies, the XRD pattern (Figure S16) and FESEM micrograph (Figure S17) of the Bi_2_O_2_S/NiTiO_3_ photoanode after degradation of SMX show that the characteristic diffraction peaks and morphology of Bi_2_O_2_S/NiTiO_3_ were maintained, thus suggesting the good stability of the Bi_2_O_2_S/NiTiO_3_ photoanode.

Radical trapping experiments were conducted to identify the reactive species responsible for the degradation of the Bi_2_O_2_S/NiTiO_3_ photoanode. As shown in Figure 5b, the degradation efficiency dropped from 80% in the blank experiment (without scavengers) to 12% when the hydroxyl radicals were scavenged. The trapping of photogenerated holes and superoxide anion radicals decreased the degradation efficiencies to 51 and 32%, respectively. The radical trapping results indicate that the hydroxyl radicals are the primary reactive species responsible for the degradation of sulfamethoxazole, with the superoxide anion radicals playing a secondary role and the photogenerated hole contributing around 12%. This suggests that the photogenerated holes primarily react with water molecules to generate hydroxyl radicals rather than oxidizing the pollutants directly.

### 3.5 Proposed Degradation Pathway

**Scheme 1:**
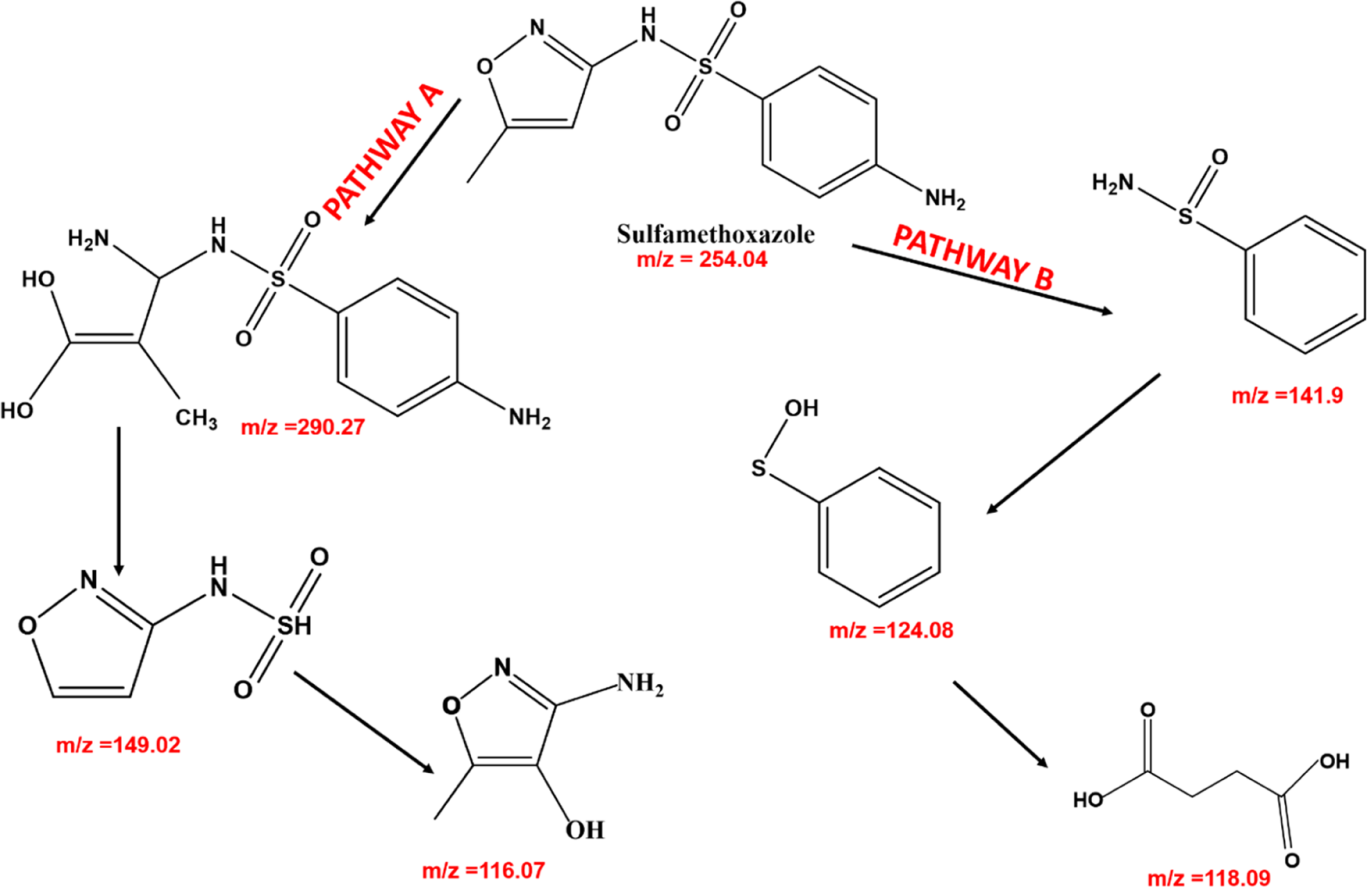
Proposed Degradation Intermediates and Byproducts

The degradation intermediates and byproducts of sulfamethoxazole over the Bi_2_O_2_S/NiTiO_3_ photoanode were investigated. Scheme 1 shows two possible pathways for the mass-to-charge ratio (*m*/*z*) observed in MS spectra (Figure S18). In pathway A, sulfamethoxazole (*m*/*z* = 254.04) undergoes oxidation to form the hydroxylated derivatives (*m*/*z* = 290.27), followed by hydrolysis leading to the cleavage of the aniline ring (*m*/*z* = 149.02), and then further oxidative breakdown, leading to desulfonation (*m*/*z* = 116.07). In contrast, pathway B suggests that sulfamethoxazole (*m*/*z* = 254.04) undergoes oxidation leading to demethylation, deamination, and cleavage of the isoxazole ring (*m*/*z* = 141.9), followed by further oxidation to remove the amino group from the sulfonamide group *(m*/*z* = 124.08), and then fragmentation and opening of the benzene ring and rearrangement (*m*/*z* = 118.09).^51−53^ The proposed degradation pathway illustrates the complex oxidative breakdown of sulfamethoxazole, highlighting the potential intermediates and final products obtained from the degradation process.

### 3.6 Proposed Z- Scheme Heterojunction Formation and the Degradation Mechanism

**Scheme 2:**
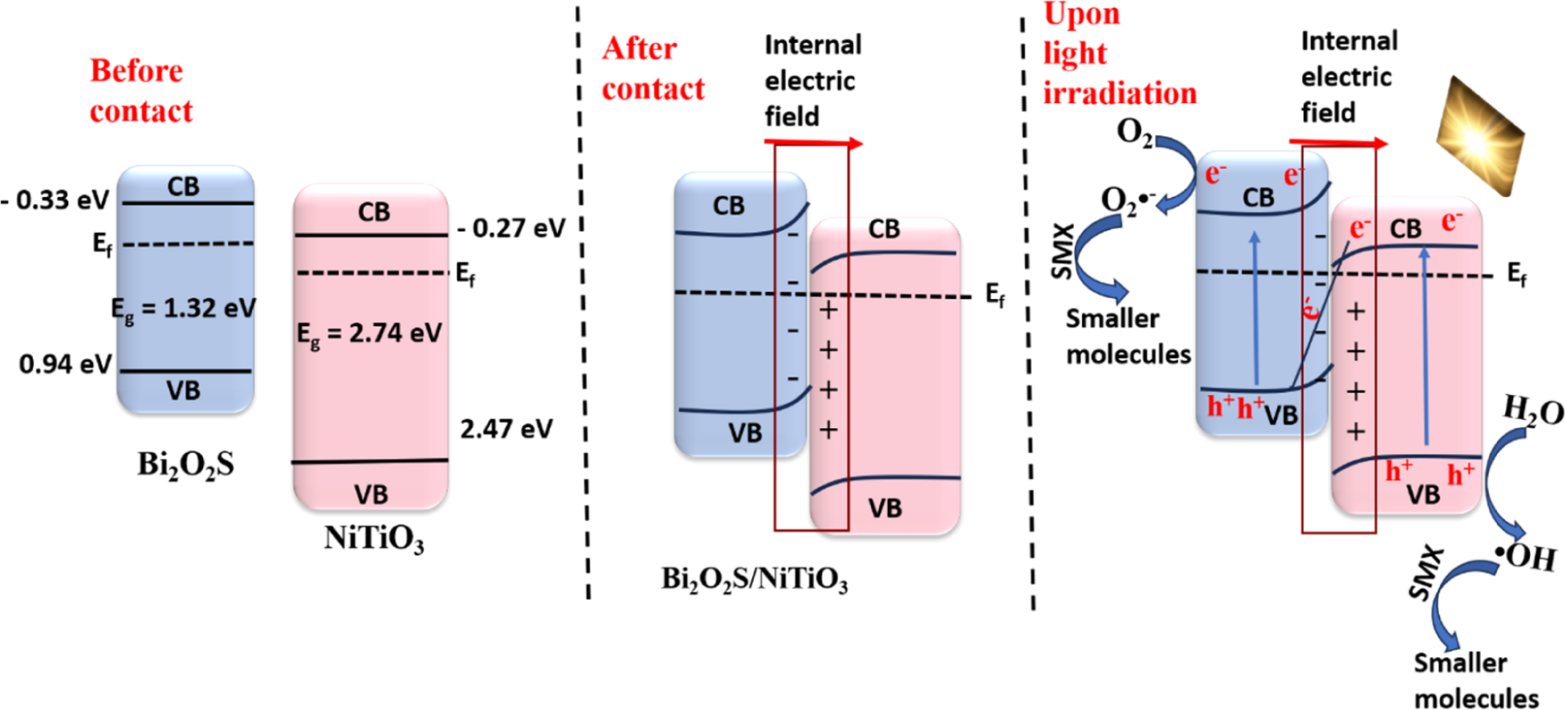
Possible Heterojunction Formation Mechanism of the Z-Scheme Bi_2_O_2_S/NiTiO_3_ Heterojunction Photoanode

The suppressed rate of recombination of the photogenerated holes (h^+^) and electrons (e^–^) in the Bi_2_O_2_S/NiTiO_3_ photoanode is evident from the photoluminescence spectra, time-resolved photoluminescence decay spectra, and photocurrent density. Considering the position of the conduction and valence bands of Bi_2_O_2_S and NiTiO_3_, as confirmed by the Mott–Schottky analysis and valence XPS spectra, two models of heterojunctions, type II and the Z-scheme, are possible. Typically, in a type II heterojunction formation, the photogenerated e^–^ in the CB of Bi_2_O_2_S will migrate to the CB of NiTiO_3_ while the photogenerated h^+^ in the VB of NiTiO_3_ will migrate to the VB of Bi_2_O_2_S.^54^ However, following the information provided in the free radical trapping experiments and XPS analysis, the type II model is inadequate for explaining the photoelectrocatalytic behavior of the Bi_2_O_2_S/NiTiO_3_ photoanode. This is because the h^+^ of Bi_2_O_2_S does not possess sufficient oxidation potential required to oxidize water molecules to generate hydroxyl radicals. Also, the e^–^ of NiTiO_3_ does not have adequate reduction potential to generate a superoxide anion radical. Hence, a suitable heterojunction formation scheme to explain the heterojunction formation between Bi_2_O_2_S and NiTiO_3_ is the Z-scheme heterojunction. As displayed in Scheme 2, before contact, the Fermi level of Bi_2_O_2_S is higher than that of NiTiO_3_; therefore, for the Fermi level to reach equilibrium, electrons will migrate from Bi_2_O_2_S to NiTiO_3_. As explained by the XPS analysis, after contact, there was an increase in the electron density in Bi_2_O_2_S, leading to electron accumulation and depletion in NiTiO_3_. This implies that when Bi_2_O_2_S and NiTiO_3_ come into contact to form a heterojunction, the band bending that occurs because of electron accumulation and depletion leads to the formation of a built-in electric field at the interface, which causes effective charge separation and transfer. Upon light illumination, there are photoexcited charge carriers (h^+^ and e^–^) in Bi_2_O_2_S and NiTiO_3_ (eq 9). The built-in electric field and band bending effect cause the e^–^ of Bi_2_O_2_S to accumulate and be retained in the CB of Bi_2_O_2_S and the h^+^ of NiTiO_3_ is confined in the VB of NiTiO_3,_ thereby causing the e^–^ in the CB of NiTiO_3_ and the h^+^ in the VB of Bi_2_O_2_S to recombine, with the interfacial S–O for easy electron transport. Therefore, the S–O bond at the interface of the heterojunction serves as a recombination center for the photogenerated holes and electrons in the valence and conduction bands with lower oxidation and reduction potentials, respectively. This ensures long-lived, useful charge carriers for improved oxidation and reduction capabilities. The preserved h^+^ of NiTiO_3_ (+2.7 eV) is used for the oxidation of water to produce hydroxyl radicals (^•^OH/H_2_O = +2.27 V) for the degradation of sulfamethoxazole (eq 10). The applied potential will then ensure that the e^–^ of Bi_2_O_2_S (−0.34 eV) is transferred to the cathode where it is used for the reduction of absorbed oxygen to generate superoxide radical anions (O_2_/O_2_^•–^ = −0.33 V), which also take part in the overall degradation process of sulfamethoxazole (eq 11), leading to the formation of smaller molecules (eq 12). The Z-scheme heterojunction formation in the Bi_2_O_2_S/NiTiO_3_ photoanode established a better photoelectrocatalytic system with improved charge transfer and effective charge separation.
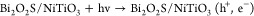
9
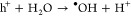
10
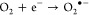
11
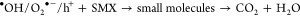
12

## 4 Conclusions

This work successfully demonstrated the fabrication and application of a Z-scheme Bi_2_O_2_S/NiTiO_3_ heterojunction photoanode for the photoelectrochemical degradation of sulfamethoxazole in water. The synthesis route led to the formation of a heterojunction, as supported by the data obtained from the various characterization techniques, explaining the strong interactions and the existence of a covalent bond (S–O) between Bi_2_O_2_S and NiTiO_3_ semiconductors. Our investigation into the properties and applications of Bi_2_O_2_S/NiTiO_3_ heterojunction photoanodes has shown their ability to harness visible light and possess charge separation when compared to pristine Bi_2_O_2_S and NiTiO_3_. Moreover, the elucidation of the proposed Z-scheme heterojunction formation and degradation mechanism provided valuable insights into the role of charge carriers in the degradation process. In summary, the Z-scheme Bi_2_O_2_S/NiTiO_3_ heterojunction photoanode is a promising solution for addressing emerging contaminants in water.

## Supporting Information Available

List of chemicals, characterization methods, and instruments used, photodegradation processes and formulas, FESEM micrographs of Bi_2_O_2_S and NiTiO_3_, XPS spectra of S 2p (Bi_2_O_2_S), FTIR spectra of Bi_2_O_2_S, NiTiO_3_ and Bi_2_O_2_S/NiTiO_3_, contact angle measurements, Tauc plot of Bi_2_O_2_S/NiTiO_3_, Mott–Schottky plots of Bi_2_O_2_S and NiTiO_3_, XPS VB of Bi_2_O_2_S and NiTiO_3_, time-resolved photoluminescence decay spectra of Bi_2_O_2_S, NiTiO_3_, and Bi_2_O_2_S/NiTiO_3,_ Nyquist circuit model, the PZC plot of Bi_2_O_2_S/NiTiO_3_, TOC graph, pseudo-second-order curve of Bi_2_O_2_S/NiTiO_3_, XRD patterns of Bi_2_O_2_S/NiTiO_3_ before and after degradation, FESEM micrograph of Bi_2_O_2_S/NiTiO_3_ after degradation, and UPLC-MS spectra. (PDF)
