# Supplementary material for: Interfacial Engineering of a Z-Scheme Bi2O2S/NiTiO3 Heterojunction Photoanode for the Degradation of Sulfamethoxazole in Water
Source: ACS Appl Mater Interfaces. 2024 Dec 5;17(1):1385–98. doi: 10.1021/acsami.4c20102 (PMC11783549; doi:10.1021/acsami.4c20102)
Supplement: Supplementary file 1 — am4c20102_si_001.pdf [file am4c20102_si_001.pdf]

## SUPPORTING INFORMATION

# Interfacial engineering of a Z-scheme $Bi_2O_2S/NiTiO_3$ heterojunction photoanode for the degradation of sulfamethoxazole in water

Kehinde D Jayeola[a,b], Dimpo S Sipuka[a,b], Tsholofelo I. Sebokolodi[a,b], Jonathan O. Babalola[c,d], Minghua Zhou[e], Frank Marken[a,f], Omotayo A. Arotiba[a,b,*]

[a] Department of Chemical Sciences, University of Johannesburg, Doornfontein, Johannesburg, 2028, South Africa

[b] Centre for Nanomaterials Science Research, University of Johannesburg, Johannesburg, 2028, South Africa

[c] Department of Chemistry, University of Ibadan, Ibadan, Oyo state, 200005, Nigeria

[d] Bowen University, Iwo, Osun State, 232101, Nigeria

[e] Tianjin Key Laboratory of Environmental Technology for Complex Trans-Media Pollution, College of Environmental Science and Engineering, Nankai University, Tianjin 300350, China

[f] Department of Chemistry, University of Bath, Claverton Down, Bath, BA2 7AY UK

Corresponding Author: oarotiba@uj.ac.za

## Chemicals used

Nickel acetate tetrahydrate, acetic acid, ethylene glycol, tetra-n-butyl ortho-titanate, thiourea, lithium monohydrate, polyvinylidene fluoride (PVDF), N-methyl-2-pyrrolidone (NMP), potassium ferrocyanide ($K_4[Fe(CN)_6]$), tert-butanol, acrylamide, sodium ethylenediaminetetraacetate (EDTA), and sodium sulphate ($Na_2SO_4$).

## Characterisation of Photoanodes

Instrumentation and methods are those reported previously [1]. The composition and crystallinity of the fabricated photoanode materials were analyzed by powder X-ray diffraction (XRD) using a Rigaku Ultima IV diffractometer from Japan. The measurements were conducted with Cu Kα radiation (k = 0.15406) and a K-beta filter, operating at 30 mA and 40 kV. Surface valence, elemental compositions, and electronic structure of the photoanode materials were evaluated using X-ray photoelectron spectroscopy (XPS) on a Thermo Scientific ESCALAB 250Xi spectrometer, equipped with a monochromatic Al-Kα X-ray source (1486.7 eV) at 300 W.

The morphology of the photoanode materials was investigated using a field-emission scanning electron microscope (FE-SEM, JEOL JSM-7500F, Japan) and transmission electron microscopy (TEM, JOEL, Germany). UV-vis diffuse reflectance spectrophotometry (DRS) on a Cary 60 UV–vis spectrophotometer (Malaysia) was utilized to examine the light absorption properties. Optical properties and the recombination rate of photogenerated holes and electrons were studied using photoluminescence (PL) spectroscopy (F-186 2710, HITACHI, Japan). The electric charge on the surface of the fabricated materials was measured through Zeta potential analysis using a ZEECOM ZC-3000 instrument from Japan. The extent of mineralisation of sulfamethoxazole was monitored using a total organic carbon (TOC) analyser (Teledyne Tekmar TOC fusion). The contact angles of the semiconductors were calculated using contact angle analyser (Biolin Scientific).

Photoelectrochemical measurements, including electrochemical impedance spectroscopy (EIS), photocurrent response, and Mott-Schottky measurements, were conducted using an Autolab PGSTAT204 potentiostat/galvanostat (Netherlands) employing a three-electrode setup. The fabricated electrodes served as the working electrode, while a platinum wire functioned as the counter electrode, and an Ag/AgCl (3 M KCl) electrode was employed as the reference electrode. The photoanode was exposed to irradiation from a 100 W solar simulator (Oriel LCA-100 model, USA), with a light density of 0.1 W/cm². For EIS, measurements were performed in a solution containing a 5 mM $[Fe(CN)_6]^{3/4-}$ in 0.1 M KCl with a +0.25 V vs. Ag/AgCl applied potential, covering a frequency range from 100 kHz to 0.1 Hz. Mott-Schottky measurements were recorded in the absence of light using a 5 mM $[Fe(CN)_6]^{3/4-}$ in 0.1 M KCl solution as the electrolyte. Photocurrent response data were evaluated under both dark and light conditions using a 0.1 M $Na_2SO_4$ solution, with an applied potential of 1.5 V vs. Ag/AgCl.

The degradation pathway of the PEC degradation was investigated using UPLC-MS, (WATERS, USA). An optimised column temperature of 60 °C was used for the chromatographic separation, which was performed on a Waters HSS T3 C18 analytical column measuring 150 mm x 2.1 mm and containing 1.8 µm particles. The injection volume was 10 µL and the analysis time was 10 minutes. To aid in the detection of ESI-compatible compounds, the SYNAPT G1 mass spectrometer was used under multiple reaction monitoring modes with both positive and negative electrospray ionization. A 2.5 KV capillary voltage was applied, and a 0.1 s scan time covering a mass range of 50-1200 Daltons was used. The source temperature and desolvation temperature

were set to 120°C and 450°C, respectively. Argon was used as the collision gas, while nitrogen served as the desolvation and nebulizer gas. The target compounds were identified based on the matching of their chromatography retention time, and the instrumental control, data acquisition, and processing were carried out using MassLynx V4.1 (Waters, USA). To aid in the detection of ESI-compatible compounds, the SYNAPT G1 mass spectrometer was used under multiple reaction monitoring modes with both positive and negative electrospray ionization. A 2.5 KV capillary voltage was applied, and a 0.1 s scan time covering a mass range of 50-1200 Daltons was used. The source temperature and desolvation temperature were set to 120°C and 450°C, respectively. Argon was used as the collision gas, while nitrogen served as the desolvation and nebulizer gas. The target compounds were identified based on the matching of their chromatography retention time, and the instrumental control, data acquisition, and processing were carried out using MassLynx V4.1 (Waters, USA).

**Photoelectrochemical degradation experiments**

In a two-electrode system, the $Bi_2O_2S/NiTiO_3$ photoanode as the working electrode and a platinum sheet was used as the cathode, the degradation experiments of the $Bi_2O_2S/NiTiO_3$ photoanode were conducted using a in a 50 mL quartz reaction cell containing 5 mg/L of sulfamethoxazole in 0.1 M $Na_2SO_4$. With an anode-cathode spacing of 3 cm, the photoanode was irradiated under a 100 W Xenon Lamp and the degradation efficiency of the pristine photoanodes and the composite photoanode were compared. The effects of pH and current density on the photoelectrochemical performance of the PEC process were examined. And, aliquots of the samples are collected at different time intervals using a disposable syringe and analysed using a UV-vis spectrophotometer.

The degradation efficiency of $Bi_2O_2S/NiTiO_3$ photoanode was calculated using the formula Equation S1:

$$\text{Percentage degradation efficiency} = \frac{C_o - C_t}{C_o} * 100 \qquad \text{Eq. S1}$$

Where $C_o$= Initial concentration and $C_t$ = Concentration at time t.

The percentage removal of total organic carbon in the synthetic sulfamethoxazole was obtained with the formula in Equation S2

$$\text{Percentage extent of mineralisation (\%)} = \frac{TOC_o - TOC_t}{TOC_o} * 100 \qquad \text{Eq. S2}$$

Where $TOC_o$= Initial value before treatment and $TOC_t$ = Final TOC after treatment at a particular time.

The main reactive species responsible for the degradation of sulfamethoxazole over $Bi_2O_2S/NiTiO_3$ photoanode via a free radical quenching experiment. Hydroxyl radicals, superoxide radicals, and photogenerated holes were trapped using 2 mM tert-butanol, 2 mM acrylamide, and 2 mM sodium ethylenediaminetetraacetate (EDTA) respectively.

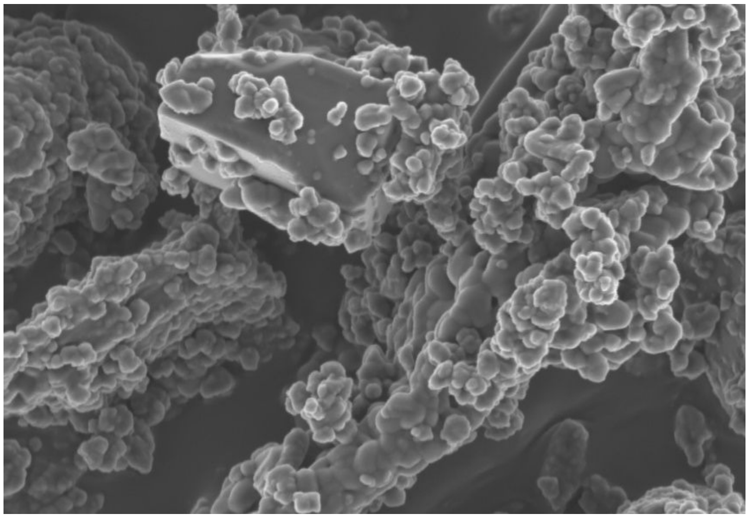

Figure S1. FESEM micrograph of $NiTiO_3$

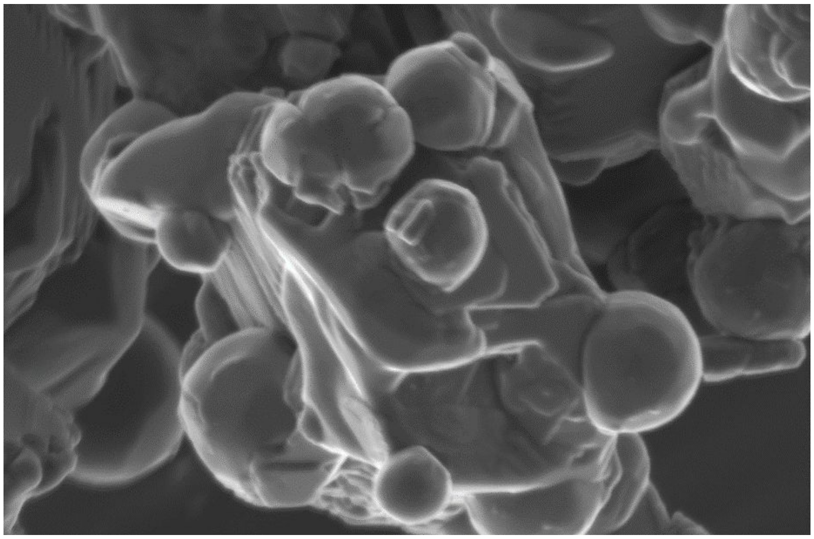

Figure S2. FESEM micrograph of $Bi_2O_2S$

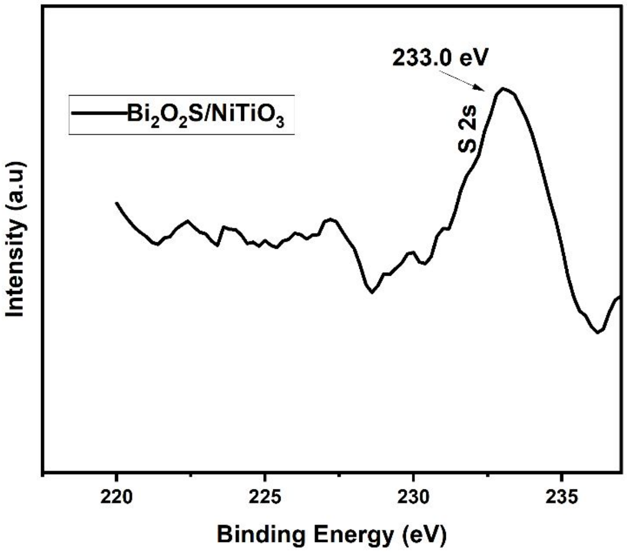


Figure S3.XPS spectra of S 2p of $Bi_2O_2S/NiTiO_3$

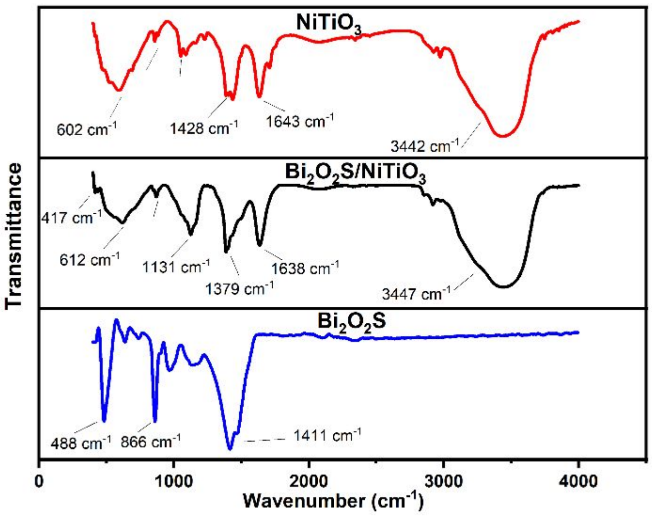


Figure S4: FTIR spectra of $Bi_2O_2S$, $NiTiO_3$ and $Bi_2O_2S/NiTiO_3$

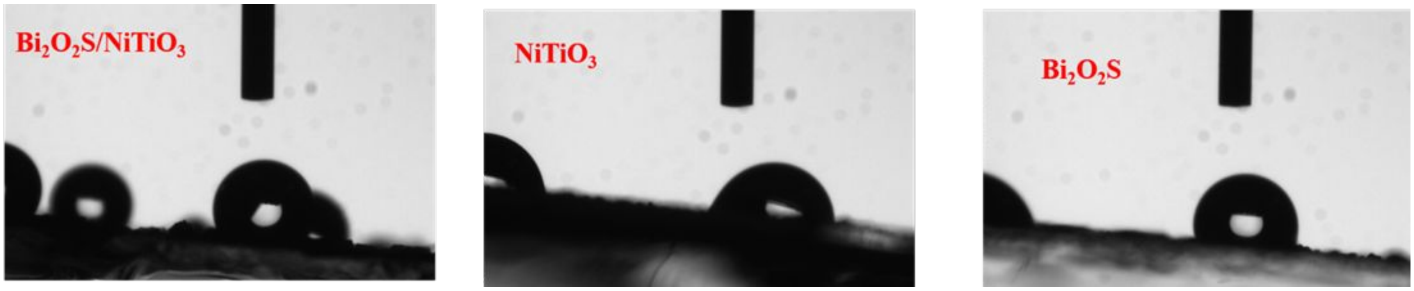


Figure S5. Contact angle of $Bi_2O_2S$, $NiTiO_3$ and $Bi_2O_2S/NiTiO_3$ photoanodes

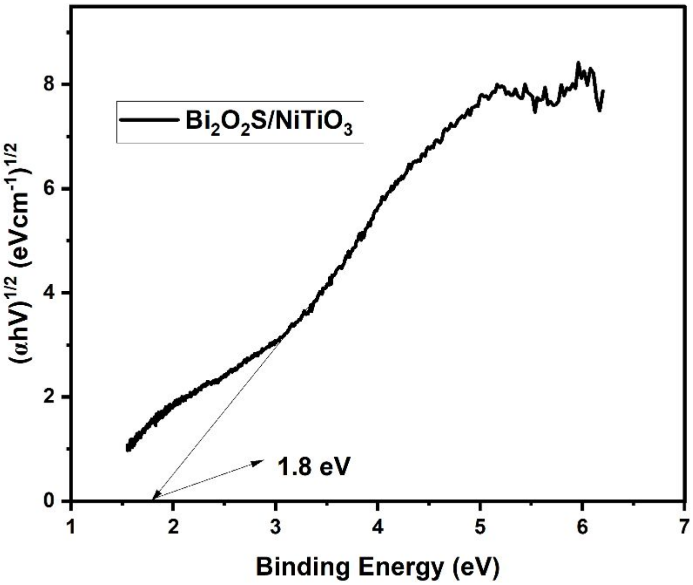


Figure S6. Tauc plot of $Bi_2O_2S/NiTiO_3$

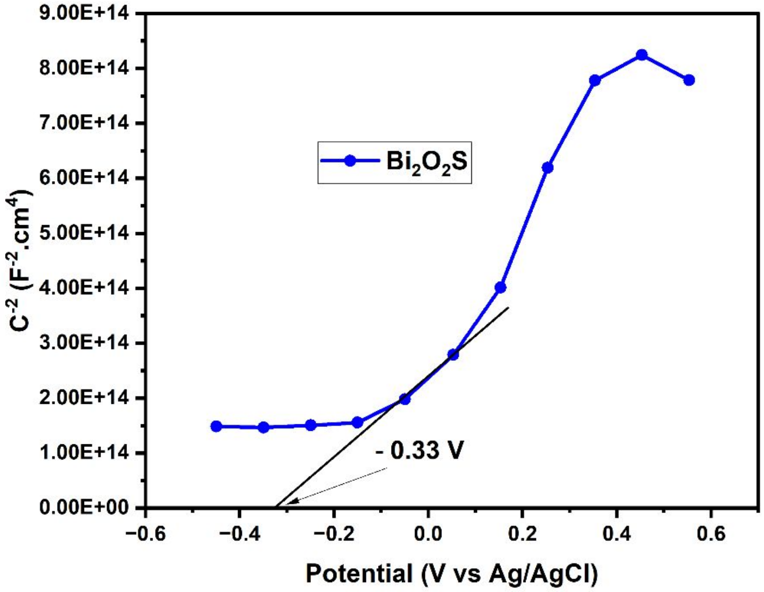


Figure S7. Mott Schottky curve of $Bi_2O_2S$

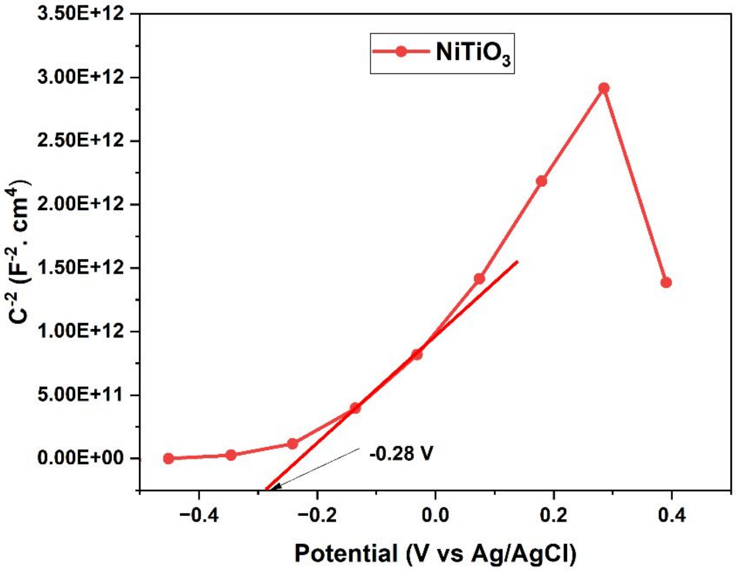


Figure S8. Mott Schottky curve of $NiTiO_3$

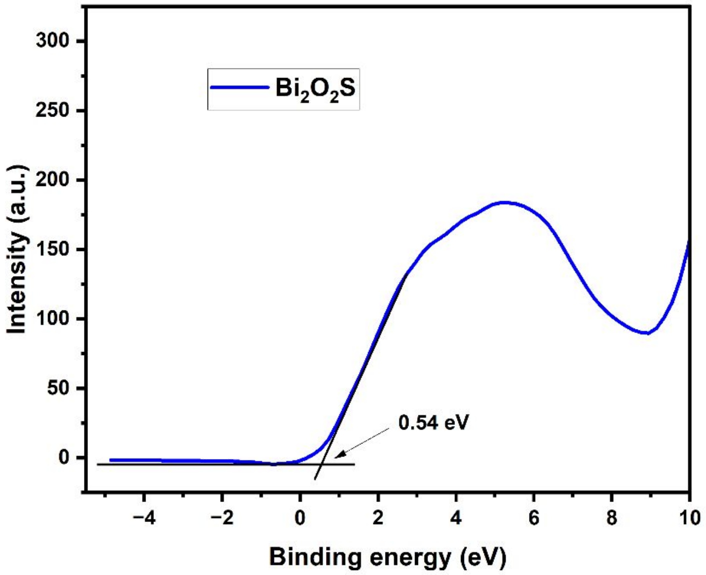


Figure S9: XPS valence spectra of $Bi_2O_2S$

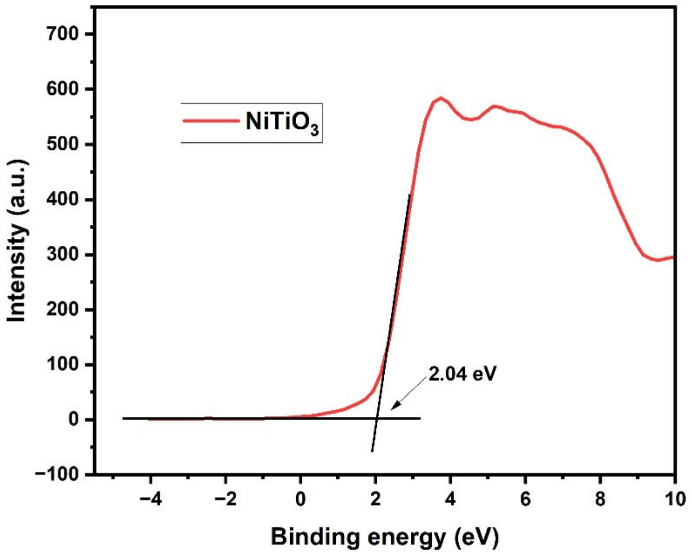


Figure S10: XPS valence spectra of $NiTiO_3$

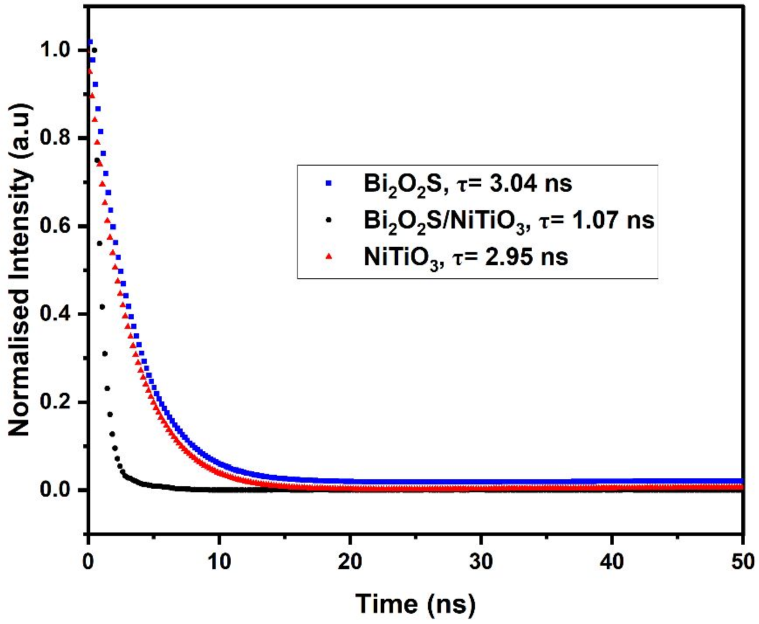


Figure S11. Time resolved photoluminescence decay spectra of $Bi_2O_2S$, $NiTiO_3$ and $Bi_2O_2S/NiTiO_3$

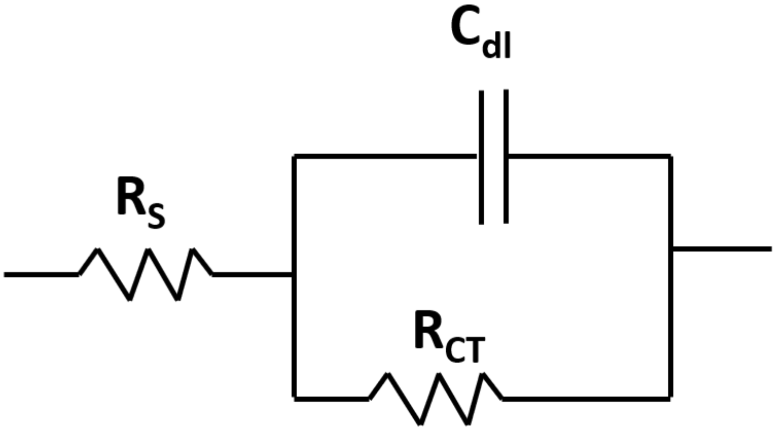


Figure S12. Equivalent circuit model for $Bi_2O_2S$, $NiTiO_3$ and $Bi_2O_2S/NiTiO_3$

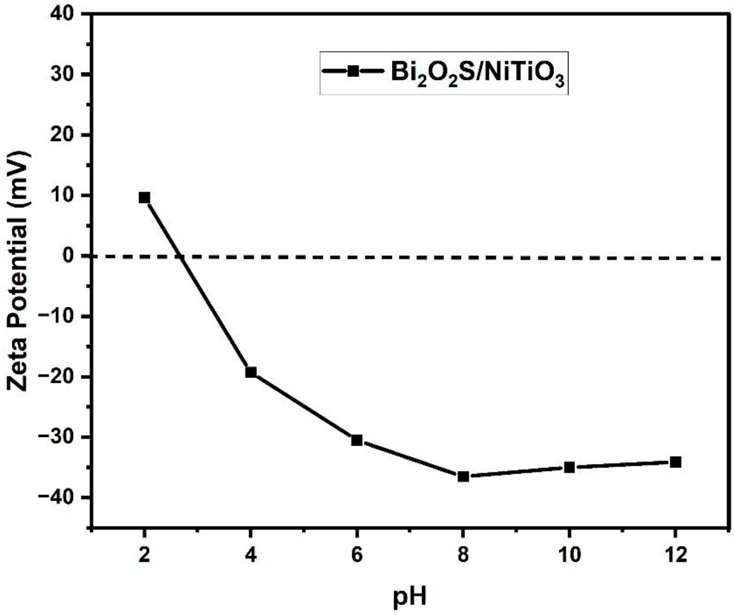


Figure S13: PZC plot of $Bi_2O_2S/NiTiO_3$

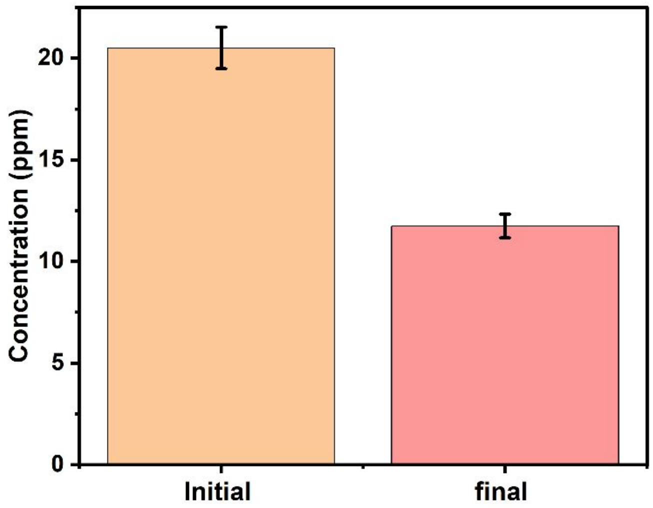


Figure S14. Percentage removal of total organic carbon of $Bi_2O_2S/NiTiO_3$

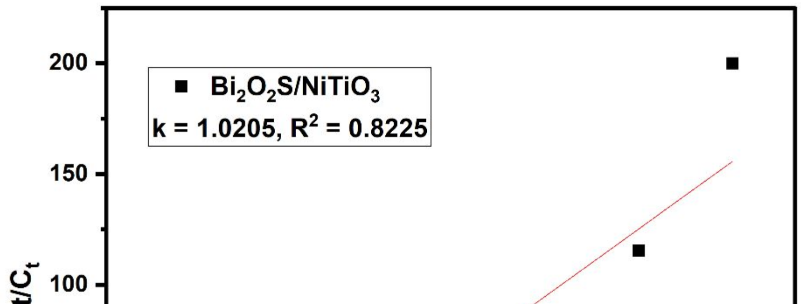

Figure S15. Pseudo second order plot of $Bi_2O_2S/NiTiO_3$

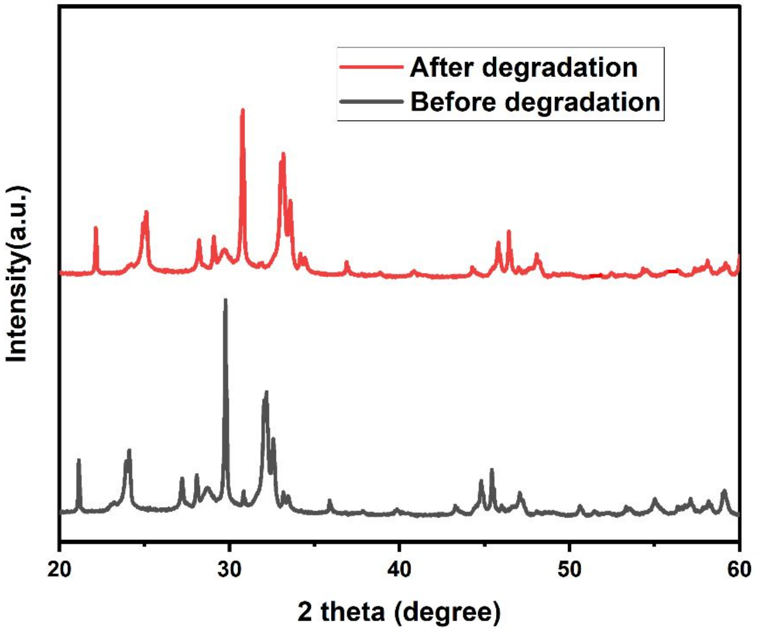


Figure S16. XRD pattern of $Bi_2O_2S/NiTiO_3$ before and after degradation process.

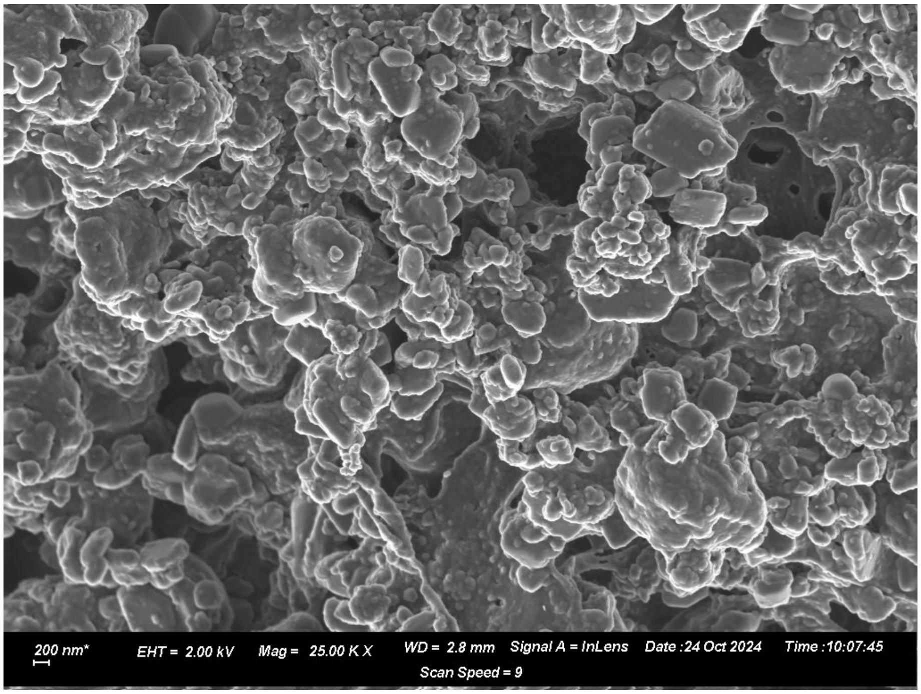


Figure S17. FESEM micrograph of $Bi_2O_2S/NiTiO_3$ after degradation process.

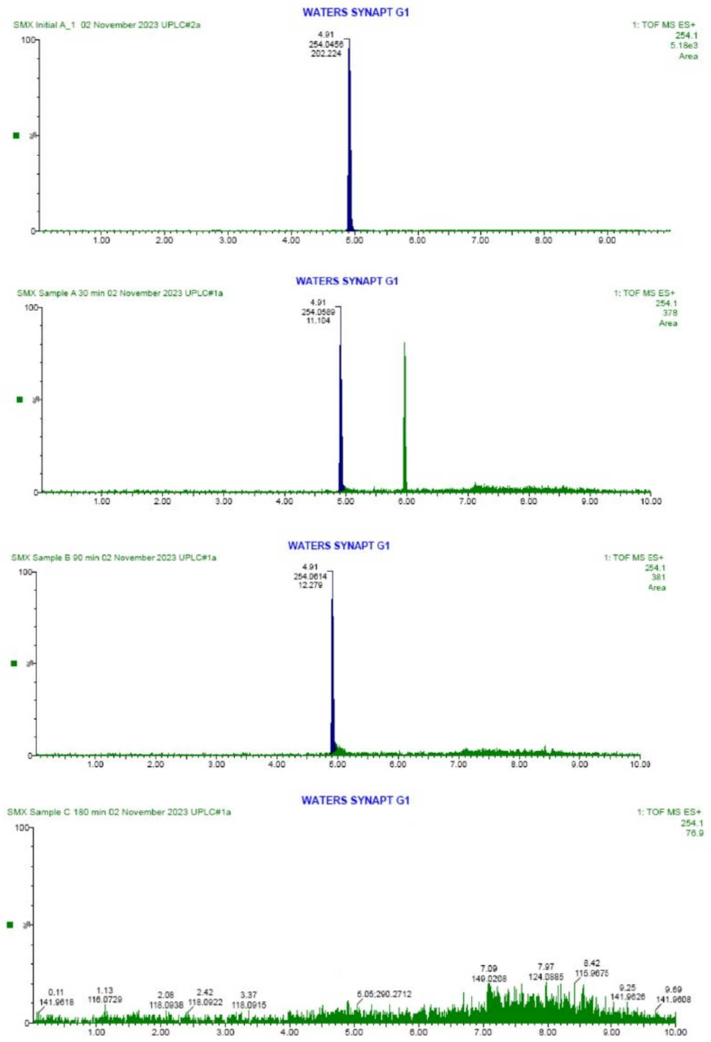


Figure S18. UPLC-MS spectra of the degradation of SMX $Bi_2O_2S/NiTiO_3$ photoanode

## References

[1] K.D. Jayeola, D.S. Sipuka, T.I. Sebokolodi, O.V. Nkwachukwu, C. Muzenda, B.A. Koiki, J.O. Babalola, M. Zhou, O.A. Arotiba, The design and characterisation of a Z-scheme Bi2O2S/ZnO heterojunction photoanode for the photoelectrochemical removal of ciprofloxacin in synthetic and real wastewater, Chemical Engineering Journal, 479 (2024) 147482.
